# Context-Dependent Effects of Maca Extracts on Signaling, Apoptosis, and Lipid Metabolism Markers in Prostate Cancer Mono- and Co-Culture Models

**DOI:** 10.3390/cells15121090

**Published:** 2026-06-16

**Authors:** Adam Jan Olichwier, Magdalena Bruzgo-Grzybko, Izabela Suwda Kalita, Aleksandra Golonko, Natalia Bielicka, Ewa Chabielska, Anna Gromotowicz-Poplawska

**Affiliations:** 1 Radiopharmacy Centre, Medical University of Bialystok, 15-569 Bialystok, Poland; izabela.kalita@umb.edu.pl (I.S.K.); anna.gromotowicz-poplawska@umb.edu.pl (A.G.-P.); 2 Department of Biopharmacy and Radiopharmacy, Medical University of Bialystok, 15-222 Bialystok, Poland; magdalena.bruzgo-grzybko@umb.edu.pl (M.B.-G.); natalia.marcinczyk@umb.edu.pl (N.B.); ewa.chabielska@umb.edu.pl (E.C.); 3 Clinical Research Center, Medical University of Bialystok, 15-276 Bialystok, Poland; aleksandra.golonko@umb.edu.pl

**Keywords:** doxorubicin, prostate cancer, tumor microenvironment, lipid metabolism, signaling pathways, co-culture

## Abstract

**Highlights:**

**What are the main findings?**

- Maca extracts exhibit limited cytotoxicity but significantly modulate signaling, apoptosis, and lipid metabolism in prostate cancer cells in a model-dependent manner.
- Co-culture with fibroblasts was associated with reduced doxorubicin sensitivity and altered lipid metabolism-related responses.

**What are the implications of the main findings?**

- Stromal interactions substantially influenced cellular responses to treatment under co-culture conditions and can mask or alter drug effects observed in mono-culture.
- Maca acts primarily as a microenvironment-dependent modulator of metabolic and signaling pathways, highlighting context-dependent effects on signaling- and metabolism-related markers rather than direct cytotoxic activity.

**Abstract:**

Prostate cancer progression and therapy response are strongly influenced by the tumor microenvironment (TME), particularly stromal fibroblasts that regulate survival signaling, metabolism, and drug resistance. In this study, we investigated whether extracts from three *Lepidium meyenii* (maca) morphotypes, yellow (MY), red (MR), and black (MB), modulate doxorubicin (DOX) responses in 22Rv1 prostate cancer cells under mono-culture and co-culture conditions with human dermal fibroblasts (HDFa). Cell viability, proliferation, apoptosis-related proteins, lipid droplets (LDs) accumulation, and selected signaling markers were analyzed. In mono-culture, maca extracts exhibited limited cytotoxicity, with MB showing the strongest but still moderate effect. Co-treatment with DOX did not enhance cytotoxicity and resulted in context-dependent modulation of caspase-3 and caspase-8. In co-culture, HDFa cells reduced DOX sensitivity, suggesting altered treatment responses under co-culture conditions. Morphometric analysis suggested fibroblast activation-like changes. Across models, maca reduced LDs accumulation, while increased adipose triglyceride lipase (ATGL) levels in co-culture suggested altered lipid utilization. Additionally, maca extracts modulated PI3K, PSMA, FOXO1, FAP, and HAT1 in a morphotype-dependent manner. Overall, maca extracts acted primarily as context-dependent modulators of signaling and lipid metabolism markers rather than direct cytotoxic agents with their effects strongly dependent on both extract type and microenvironmental context.

## 1. Introduction

Prostate cancer is one of the most frequently diagnosed malignancies in men and a leading cause of cancer-related mortality worldwide [1,2]. Although early-stage disease can often be effectively treated, advanced prostate cancer remains challenging due to therapy resistance, tumor heterogeneity, and complex molecular mechanisms that support tumor progression [3].

Prostate cancer progression involves multiple signaling pathways involved in proliferation, apoptosis, metabolism, and stromal interactions [3,4]. Prostate-specific membrane antigen (PSMA) is highly upregulated in malignant prostate tissue and is associated with tumor aggressiveness and poor clinical outcomes [5,6]. In addition, recent reviews have highlighted its relevance within the broader context of prostate tumor–stroma interactions [7]. Among intracellular pathways, the phosphoinositide 3-kinase/protein kinase B (PI3K/AKT) signaling axis is linked to cell survival, altered metabolism, and resistance to apoptosis [4,8,9]. Its downstream effector, forkhead box O1 (FOXO1), functions as a tumor suppressor regulating apoptosis and oxidative stress responses [4,9]. In addition, epigenetic regulators such as histone acetyltransferase 1 (HAT1) have emerged as important modulators of chromatin organization, gene expression, and cancer cell proliferation, linking metabolic status with transcriptional control [10].

Tumor progression is further shaped by the TME [3], where cancer-associated fibroblasts (CAFs) are known to contribute to extracellular matrix remodeling, invasion, metabolic support, and therapy resistance [11]. Recently, Lupsa et al. (2026) [7] provided a comprehensive overview of the functions of CAFs, heterogeneity, and resistance mechanisms in prostate cancer. Fibroblast activation protein (FAP), a marker of activated fibroblasts, plays a key role in these processes [11,12,13]. Conventional mono-culture models, such as 22Rv1 cells grown as a monolayer, do not capture these interactions, whereas co-culture systems with fibroblasts (e.g., human dermal fibroblasts; HDFa) provide a simplified model of tumor–stroma interactions, which can significantly alter cancer cell proliferation, apoptosis, and signaling responses [14,15].

Altered lipid metabolism is another important feature of cancer progression [3,9]. In prostate cancer, accumulation of lipid droplets (LDs) supports energy homeostasis and contributes to therapy resistance [16,17]. Lipid metabolism is regulated by enzymes such as adipose triglyceride lipase (ATGL) and its co-activator α/β-hydrolase domain-containing protein 5 (ABHD5), which control triglyceride hydrolysis [18,19] and are functionally linked to signaling pathways including PI3K/AKT [9,18]. LD-associated proteins, such as perilipin-3 (PLIN3), further regulate lipid storage and mobilization, contributing to altered lipid metabolism under stress conditions [16,19].

Doxorubicin (DOX), a widely used chemotherapeutic agent, induces cytotoxicity through DNA damage and oxidative stress; however, its efficacy is limited by resistance mechanisms, often associated with altered pro-survival signaling and metabolic adaptation [3,9]. Increasing evidence suggests that both the TME and altered metabolic responses play key roles in shaping treatment response [14,15,20].

Therefore, there is growing interest in natural compounds as modulators of cellular and metabolic responses. *Lepidium meyenii* (maca) is a plant with reported antioxidant, anti-inflammatory, and metabolic regulatory properties, as well as reported biological activity in cancer-related models [21,22]. It occurs in different morphotypes, yellow (MY), red (MR), and black (MB), which differ in phytochemical composition and biological activity [21,22,23,24]. These differences likely reflect variations in bioactive compounds such as macamides, glucosinolates, and polyphenols, which may influence their biological effects [22,24]. Previous studies have reported that maca extracts exhibit antioxidant, anti-inflammatory, and metabolic regulatory properties and have been shown to reduce prostate size in vivo, suggesting effects related to proliferation control in prostate tissue [25]. Moreover, plant-derived compounds have been shown to modulate lipid metabolism and intracellular signaling pathways associated with cancer progression, including PI3K/AKT-mediated mechanisms, potentially influencing therapeutic responses [26].

Therefore, the aim of this study was to investigate the effects of different maca morphotypes (MY, MR, MB) on DOX-induced responses in 22Rv1 prostate cancer cells. We focused on key regulators of proliferation, apoptosis, and lipid metabolism, and by comparing mono-culture and co-culture with HDFa fibroblasts, we aimed to evaluate how stromal interactions under co-culture conditions influence cellular responses. We hypothesized that maca extracts would be associated with morphotype-dependent changes in signaling and lipid metabolism responses under mono- and co-culture conditions. The 22Rv1 cell line was selected because it represents an androgen receptor-positive prostate cancer model with resistance-associated features and active PI3K/AKT signaling relevant to stress adaptation and treatment response. In addition, 22Rv1 cells are commonly used to investigate apoptosis, metabolic adaptation, and therapy resistance in prostate cancer models.

## 2. Materials and Methods

### 2.1. Reagents

Primary antibodies against fibroblast activation protein (FAP; EPR20021) and goat anti-rabbit HRP-conjugated secondary antibody (ab97051) were obtained from Abcam (Cambridge, UK). GAPDH (4G5) and HRP-conjugated rat anti-mouse secondary antibody (LO-ME-3) were purchased from Bio-Rad (Hercules, CA, USA). Antibodies against adipose triglyceride lipase (ATGL; #2138), caspase-3 (CASP3; #9662), and phosphoinositide 3-kinase (PI3K; #4255) were obtained from Cell Signaling Technology (Hertfordshire, UK). Antibodies against abhydrolase domain-containing protein 5 (ABHD5; sc-100468), caspase-8 (CASP8, sc-81656), histone acetyltransferase 1 (HAT1, sc-390562), and perilipin-3 (PLIN3; sc-390968) were obtained from Santa Cruz Biotechnology (Santa Cruz, CA, USA). Forkhead box protein O1 (FOXO1, ZRB1862-25UL) was obtained from Merck (Darmstadt, Germany). The antibody against prostate-specific membrane antigen (PSMA; #37-3900) was purchased from Thermo Fisher Scientific (Grand Island, NY, USA). Unless otherwise specified, additional reagents were obtained from Sigma-Aldrich (St. Louis, MO, USA).

### 2.2. Maca Extract Preparation

Maca (*Lepidium meyenii*) is cultivated in high-altitude Andean regions, with three maca morphotypes: yellow (MY), red (MR), and black (MB), which were analyzed. Powdered root material originating from Peru was obtained via a Polish distributor (NANGA Przemysław Figura, Blękwit, Poland; MY 539, MR 010402240RG, MB MNPO-241000279). Stock suspensions were prepared in dimethyl sulfoxide (DMSO; J66650.AE, Thermo Fisher Scientific) at 10 mg/mL and filtered through 0.45 µm membranes. Working concentrations (10–500 µg/mL) were prepared in conditioned culture medium containing 1% fetal bovine serum (FBS; F7524, Sigma-Aldrich).

### 2.3. Cell Culture and Treatments

The human prostate cancer cell line 22Rv1 (CRL-2505; ATCC, Manassas, VA, USA) was used as an in vitro model of prostate carcinoma. Cells were cultured in RPMI-1640 medium (30-2001, ATCC) supplemented with 10% FBS (F7524, Sigma-Aldrich) and Antibiotic–Antimycotic Solution (100×; A5955, Merck) containing penicillin, streptomycin, and amphotericin B. Cultures were maintained at 37 °C in a humidified atmosphere with 5% CO_2_, and the medium was replaced every 48 h.

Human dermal fibroblasts, adult (HDFa; PCS-201-012, ATCC) were cultured separately in Fibroblast Basal Medium (PCS-201-030, ATCC) supplemented with L-glutamine (7.5 mM), recombinant human fibroblast growth factor (5 ng/mL), ascorbic acid (50 µg/mL), hydrocortisone hemisuccinate (1 µg/mL), recombinant human insulin (5 µg/mL), FBS (2%) (Fibroblast Growth Kit–Low Serum; PCS-201-041, ATCC), and Antibiotic–Antimycotic Solution (100×; final concentration 1%; A5955, Merck) under the same incubation conditions.

To model selected tumor–stromal interactions under co-culture conditions, co-culture experiments were performed using 22Rv1 prostate cancer cells and HDFa fibroblasts in a direct co-culture system, allowing both physical contact and paracrine signaling between the two cell types. 22Rv1 cells were seeded first and allowed to attach for 24 h, after which HDFa fibroblasts were added to initiate the co-culture. Approximately 6 × 10^6^ 22Rv1 cells (≈1.1 × 10^5^ cells/cm^2^) were seeded per 100 mm culture dish, followed by the addition of 1 × 10^6^ HDFa fibroblasts (≈1.8 × 10^4^ cells/cm^2^) after 24 h. The co-culture was then maintained for an additional 24 h prior to treatment.

For experimental treatments, cultures were transferred to medium containing 1% FBS and exposed to doxorubicin hydrochloride (DOX; D1515, Sigma-Aldrich) at concentrations of 0.5–10 µM or to maca extracts (MY, MR, MB) at 10–500 µg/mL for 24 or 48 h. For combination experiments, cells were treated with DOX at IC_25_ or IC_50_ concentrations together with MY, MR, or MB extracts (10–500 µg/mL) for 24 h. For co-cultures, 22Rv1 cells were cultured for 24 h, followed by 24 h of co-culture with HDFa fibroblasts, after which cells were treated with DOX and/or maca extracts for 24 h. Control cultures received medium containing 1% FBS with the corresponding DMSO concentration used in treated samples. For wound healing and Western blot analyses, maca extracts were used at 100 µg/mL because this concentration represented a biologically active but non-severely cytotoxic condition across the tested morphotypes. This allowed evaluation of protein expression and wound closure changes while minimizing excessive loss of viability.

### 2.4. Morphometric Analysis

Morphometric analysis of fibroblast-like cells was performed on phase-contrast images of 22Rv1-HDFa co-cultures using ImageJ software version 1.54 (NIH, Bethesda, MD, USA). Elongated, spindle-shaped cells were manually identified and selected. Cell length (major axis), width (minor axis), and area were measured following image thresholding and binarization using the “Analyze Particles” function. Aspect ratio (L/W) was calculated to assess cell elongation. Cell orientation was defined as the angle of the major axis relative to the horizontal axis, and alignment was evaluated based on the distribution of orientation angles. Nearest neighbor distance (NND) was calculated as the distance between the centroids of each cell and its closest neighboring cell within the same field. At least 20 cells per condition were analyzed from three independent experiments. Data are presented as mean ± SD and were subjected to statistical analysis.

### 2.5. Cell Viability Assay

Cell viability was evaluated using the CellTiter-Blue assay (G8080, Promega, Madison, WI, USA) according to the manufacturer’s protocol. Cells were seeded in 96-well plates at densities resulting in approximately 70% confluence and treated with DOX or maca extracts for 24 or 48 h. This assay measures the reduction of resazurin to resorufin by metabolically active cells. Absorbance was recorded using a Mobi µ2 MicroDigital microplate reader (MicroDigital Co., Seongnam-si, Gyeonggi-do, Republic of Korea) at 573 nm, with 600 nm as reference. Cell viability was expressed relative to untreated controls (100%). Dose–response curves were generated using a variable-slope model to determine IC_25_ and IC_50_ values, which were subsequently applied in combination experiments.

### 2.6. Wound Healing Assay

Wound closure under reduced-serum conditions (1% FBS) was evaluated using a wound healing assay. Cells were seeded in 6-well plates and cultured until approximately 90% confluence. A linear scratch was introduced using a sterile 200 µL pipette tip, and detached cells were removed by washing with phosphate-buffered saline (PBS). Cells were then incubated in medium containing 1% FBS with vehicle control or test compounds (DOX and selected concentrations of MY, MR, or MB). Images were captured at 0, 24, and 48 h using an Evos XL Core microscope (Invitrogen, Thermo Fisher Scientific, Waltham, MA, USA). Wound closure was quantified using the following formula:

V_T_ = (D_0_ − Dₜ)/T,

where D_0_ is the initial wound width, Dₜ represents the wound width at time T, and T is the incubation time in hours. To minimize the contribution of cell migration, assays were performed under reduced-serum conditions (1% FBS), which significantly limits migratory activity. Under these conditions, wound closure reflects combined effects of proliferation and residual migration under reduced-serum conditions.

### 2.7. Oil Red O Staining

LDs accumulation was assessed using Oil Red O (ORO) staining. Cells were rinsed with PBS and fixed with 10% neutral buffered formalin for 30 min at room temperature. Following fixation, cells were briefly incubated with 60% isopropanol. The ORO stock solution was prepared by dissolving 0.4 g ORO powder (O0625-25G, Merck) in 100 mL of 99% isopropanol and allowing the solution to equilibrate overnight. Before staining, the solution was diluted with distilled water (3:2, *v*/*v*) and filtered. Cells were stained for 15 min, rinsed with distilled water, and air-dried. Images were obtained using an Evos XL Core inverted microscope at 200× magnification.

LDs number per cell was quantified from ORO-stained images using ImageJ software version 1.54 (NIH, USA). Images were converted to 8-bit grayscale, and a consistent threshold was applied to identify ORO-positive LDs. Quantification was performed using the Analyze Particles function. Cell number was determined by manual counting within the same fields of view. Results were expressed as LDs per cell, normalized to control samples, and presented as fold change. For each experimental condition, at least three randomly selected fields of view were analyzed.

For semi-quantitative analysis, bound dye was extracted with 99% isopropanol, and absorbance was measured at 492 nm using a Mobi µ2 MicroDigital microplate reader. LDs levels were expressed relative to untreated controls.

### 2.8. Protein Quantification and Western Blot

Total protein concentration was determined using the Pierce™ 660 nm Protein Assay (22660, Thermo Fisher Scientific) with Pre-Diluted Protein Assay Standards, Bovine Serum Albumin Set (23208, Thermo Fisher Scientific) as the calibration standard.

For Western blot analysis, cells were lysed in Cell Extraction Buffer (FNN0011, Invitrogen) supplemented with 1 mM phenylmethanesulfonyl fluoride (36978, Thermo Fisher Scientific) and Halt™ Protease Inhibitor Cocktail (1862209, Thermo Fisher Scientific). Lysates were incubated on ice for 30 min and centrifuged at 15,000 rpm for 10 min at 4 °C. Equal protein amounts were mixed with 2× Laemmli buffer (161-0737, Bio-Rad), heated at 95 °C for 10 min, and separated by SDS-PAGE using 4–15% Mini-PROTEAN TGX Stain-Free gels (4568084, Bio-Rad). Proteins were transferred to PVDF membranes (Trans-Blot Turbo Mini-size LF PVDF Membrane; 10026934, Bio-Rad) using a Trans-Blot Turbo system (Bio-Rad). Membranes were blocked with EveryBlot Blocking Buffer (12010020, Bio-Rad) and incubated with primary antibodies followed by HRP-conjugated secondary antibodies. Detection was performed using Clarity Max ECL substrate (1705062, Bio-Rad), and signals were visualized with a ChemiDoc Imaging System (Bio-Rad). Protein levels were quantified densitometrically and normalized to GAPDH.

Due to the number and molecular weights of the analyzed proteins, multiple gels and membranes were required. Membranes were either analyzed separately or sectioned according to the molecular weight range of the target proteins to avoid membrane stripping and re-probing. Full-length uncropped blots together with membrane sectioning schemes and loading/transfer controls are provided in the Supplementary Materials.

### 2.9. Statistical Analysis

Statistical analyses were performed using GraphPad Prism 8.3.0 (GraphPad Software, Boston, MA, USA). Differences between groups were evaluated using one-way analysis of variance (ANOVA) followed by Tukey’s post hoc test. Data are presented as mean ± standard deviation (SD). Values of *p* < 0.05 were considered statistically significant.

## 3. Results

### 3.1. Limited Cytotoxic Effects of Maca Extracts in 22Rv1 Prostate Cancer Cells

**Figure 1:**
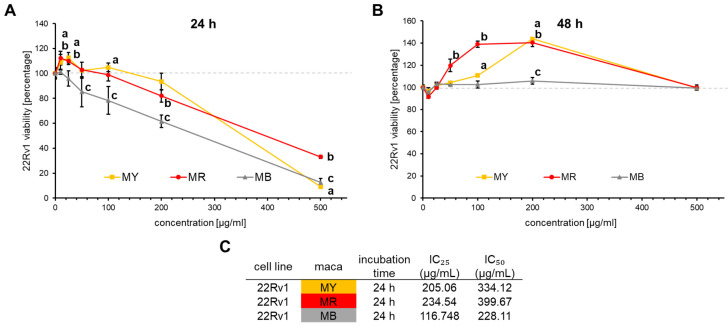
Effects of Maca Yellow (MY), Maca Red (MR), and Maca Black (MB) on the viability of 22Rv1 prostate cancer cells. Cells were treated with increasing concentrations of MY, MR, or MB for 24 h (**A**) or 48 h (**B**). (**C**) IC_25_ and IC_50_ values calculated for maca extracts after 24 h of treatment. Cell viability was assessed using the CellTiter-Blue assay. Data are expressed as mean ± SD from three independent experiments, each performed in quadruplicate (*n* = 4). The dashed line represents the viability level of the control group (100%). Statistical significance (*p* < 0.05): (a) vs. MY (0 µg/mL); (b) vs. MR (0 µg/mL); (c) vs. MB (0 µg/mL).

The effects of maca extracts on 22Rv1 cell viability were assessed after 24 and 48 h. Among the tested extracts, MB induced the most pronounced reduction in viability (Figure 1A,B). After 24 h, MY and MR showed comparable effects, with a significant decrease observed only at the highest concentration (Figure 1A). After 48 h, neither MY nor MR reduced viability; instead, a slight increase compared to control was observed (Figure 1B).

IC_25_ and IC_50_ values were calculated after 24 h exposure (Figure 1C). MB exhibited the lowest IC values, indicating the strongest effect on reducing cell viability among the tested extracts.

Overall, maca extracts exerted limited cytotoxic effects on 22Rv1 cells, with only moderate activity observed for MB, while MY and MR showed minimal impact, particularly at longer exposure times.

### 3.2. Limited Cytotoxic Effects of Combined DOX and Maca Treatment in 22Rv1 Prostate Cancer Cells

Dose–response analysis of doxorubicin (DOX) in 22Rv1 cells (Supplementary Figure S1A) allowed determination of IC_25_ and IC_50_ values (Supplementary Figure S1B). Based on these data, 0.5 µM (IC_25_) and 2.5 µM (IC_50_) DOX were selected for subsequent experiments using a 24 h incubation.

**Figure 2:**
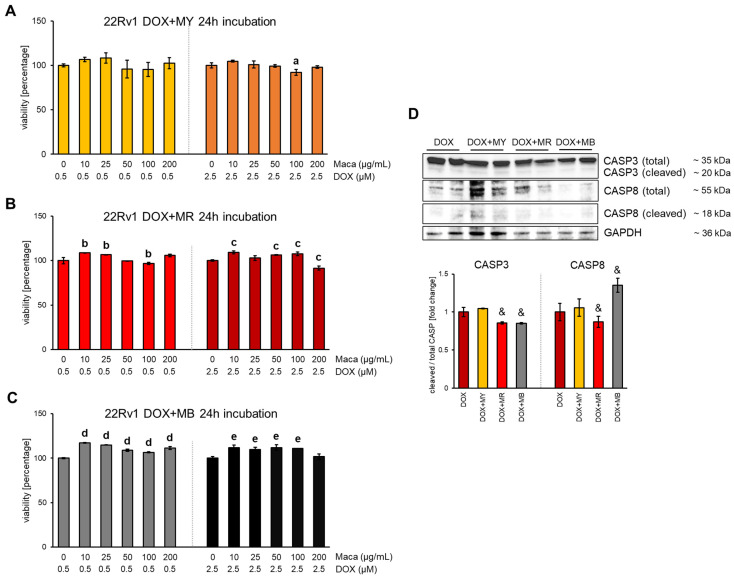
Effects of co-treatment with doxorubicin (DOX) and maca extracts on 22Rv1 cell viability and apoptosis. (**A**–**C**) Cells were co-treated with DOX (0.5 or 2.5 µM) and increasing concentrations of (**A**) Maca Yellow (MY), (**B**) Maca Red (MR), or (**C**) Maca Black (MB). Cell viability was assessed using the CellTiter-Blue assay. (**D**) Effects of DOX and maca extracts on caspase-3 (CASP3) and caspase-8 (CASP8) levels. Cells were treated with DOX (0.5 µM) in combination with maca extracts (100 µg/mL) for 24 h. Protein levels of cleaved and full-length CASP3 and CASP8 were analyzed by Western blotting. Representative immunoblots and corresponding densitometric analyses are shown. Protein levels were normalized to GAPDH. Full-length uncropped Western blots together with membrane sectioning schemes and loading/transfer controls are provided in the Supplementary Materials. Data are expressed as mean ± SD from three independent experiments. Statistical significance (*p* < 0.05): (a) vs. MY (0 µg/mL) + DOX (2.5 µM); (b) vs. MR (0 µg/mL) + DOX (0.5 µM); (c) vs. MR (0 µg/mL) + DOX (2.5 µM); (d) vs. MB (0 µg/mL) + DOX (0.5 µM); (e) vs. MB (0 µg/mL) + DOX (2.5 µM); (&) vs. DOX-treated cells.

Co-treatment with DOX and maca extracts did not result in a substantial decrease in cell viability (Figure 2A–C). MY had no significant effect at either DOX concentration (Figure 2A). MR increased viability, except at the highest tested concentrations (200 µg/mL maca and 2.5 µM DOX) (Figure 2B). A similar pattern was observed for MB, which increased viability at both DOX concentrations, with the exception of the highest maca dose (Figure 2C).

Selected apoptosis markers were analyzed following co-treatment with 0.5 µM DOX and 100 µg/mL maca (Figure 2D), including CASP8 (initiator caspase) and CASP3 (executioner caspase). MB increased CASP8 levels, MR reduced them, and MY had no effect. In contrast, CASP3 levels were decreased by MR and MB, while MY remained unchanged.

Under the tested conditions, maca extracts did not enhance DOX-induced cytotoxicity and were accompanied by differential apoptosis marker responses.

### 3.3. Differential Effects of Maca Extracts on Wound Closure in DOX-Treated 22Rv1 Cells

**Figure 3:**
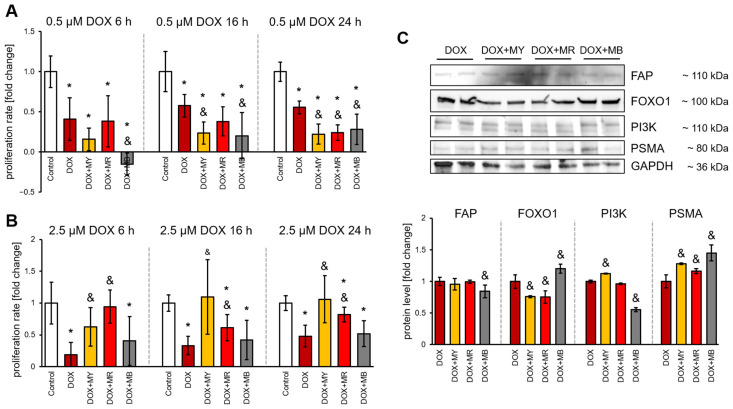
Effects of doxorubicin (DOX) and maca extracts on wound closure in 22Rv1 cells assessed by wound healing assay. Cells were treated with (**A**) 0.5 µM or (**B**) 2.5 µM DOX in combination with 100 µg/mL Maca Yellow (MY), Maca Red (MR), or Maca Black (MB) for 6, 16, and 24 h. Cells cultured in medium containing 1% fetal bovine serum (FBS) without DOX or maca extracts served as the control (Control). (**C**) Protein levels of fibroblast activation protein (FAP), forkhead box protein O1 (FOXO1), phosphoinositide 3-kinase (PI3K), and prostate-specific membrane antigen (PSMA) were analyzed after 24 h treatment with 0.5 µM DOX in combination with maca extracts. Representative immunoblots and corresponding densitometric analyses are shown. Protein levels were normalized to GAPDH. Full-length uncropped Western blots together with membrane sectioning schemes and loading/transfer controls are provided in the Supplementary Materials. Data are expressed as mean ± SD from three independent experiments. Statistical significance (*p* < 0.05): (*) vs. Control; (&) vs. DOX-treated cells.

Wound closure changes were assessed using a wound healing assay under reduced-serum conditions (1% FBS), where wound closure reflects the combined effects of proliferation and residual migration, following treatment with IC_25_ and IC_50_ concentrations of DOX (0.5 and 2.5 µM, respectively) in combination with 100 µg/mL of maca extracts (Figure 3A,B). At 0.5 µM DOX, wound closure decreased over time, with MB showing an effect at 6 h, MY and MB at 16 h, and all extracts at 24 h, compared to DOX group (Figure 3A). At 2.5 µM DOX, MY and MR increased wound closure, whereas MB did not alter wound closure compared to DOX alone (Figure 3B).

To further assess protein expression, protein levels were analyzed following co-treatment with 0.5 µM DOX and 100 µg/mL maca (Figure 3C). MY increased PI3K and PSMA levels while reducing FOXO1. MR decreased FOXO1 and increased PSMA. MB reduced FAP and PI3K levels, while increasing FOXO1 and PSMA.

Together, these data suggest that maca extracts modulated proliferation-associated responses to DOX in a morphotype-dependent manner.

### 3.4. Maca Extracts Reduce Lipid Droplets Accumulation in DOX-Treated 22Rv1 Cells

**Figure 4:**
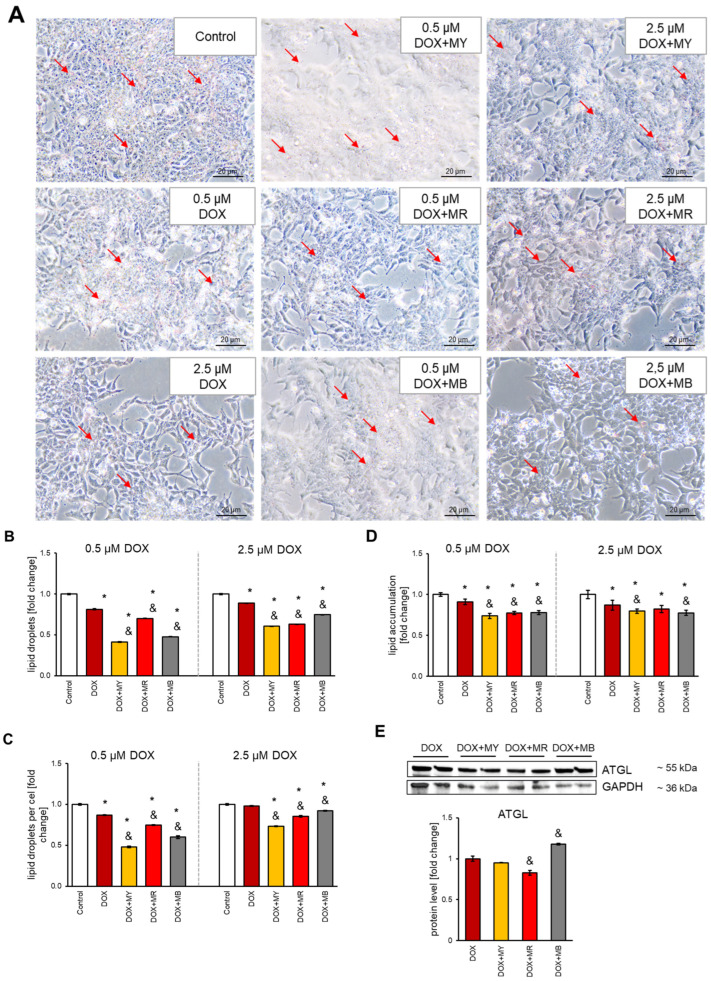
Effects of doxorubicin (DOX) and maca extracts on lipid accumulation and lipid metabolism in 22Rv1 cells. (**A**) Representative micrographs of cells treated with DOX (0.5 or 2.5 µM) alone or in combination with 100 µg/mL Maca Yellow (MY), Maca Red (MR), or Maca Black (MB), assessed by Oil Red O (ORO) staining; lipid droplets (LDs) are indicated by red arrows. (**B**–**D**) Quantification of lipid accumulation was performed as (**B**) LDs per field of view, (**C**) LDs per cell, and (**D**) intracellular lipid content measured by ORO dye extraction. (**E**) Protein levels of adipose triglyceride lipase (ATGL) were analyzed by Western blotting after treatment with DOX (0.5 µM) in combination with maca extracts. Cells cultured in medium containing 1% fetal bovine serum (FBS) without DOX or maca extracts served as the control (Control). Representative immunoblots and corresponding densitometric analyses are shown. Protein levels were normalized to GAPDH. Full-length uncropped Western blots together with membrane sectioning schemes and loading/transfer controls are provided in the Supplementary Materials. Data are expressed as mean ± SD from three independent experiments. Statistical significance (*p* < 0.05): (*) vs. Control; (&) vs. DOX-treated cells.

Given the link between proliferation and altered metabolic responses, lipid metabolism and LDs accumulation were examined. LDs were visualized using ORO staining following treatment with DOX (0.5 and 2.5 µM) and maca extracts (100 µg/mL). Representative images are shown in Figure 4A. Quantitative analysis demonstrated a reduction in LDs per field of view (Figure 4B) and per cell (Figure 4C), with the strongest effect observed for MY. This was supported by semi-quantitative analysis, based on dye extraction, which confirmed decreased intracellular lipid content in all maca-treated conditions (Figure 4D).

To further assess lipid metabolism, ATGL levels, a marker involved in lipid metabolism, were evaluated after co-treatment with 0.5 µM DOX and 100 µg/mL maca (Figure 4E). MR reduced ATGL levels, whereas MB increased it relative to DOX alone.

Reduced LD accumulation was observed across maca-treated conditions together with altered ATGL protein levels.

### 3.5. Co-Culture of 22Rv1 and HDFa

To determine whether stromal interactions influence the biological responses observed in mono-culture conditions, subsequent experiments were performed using a direct co-culture system with HDFa fibroblasts to model selected tumor–stroma interactions.

**Figure 5:**
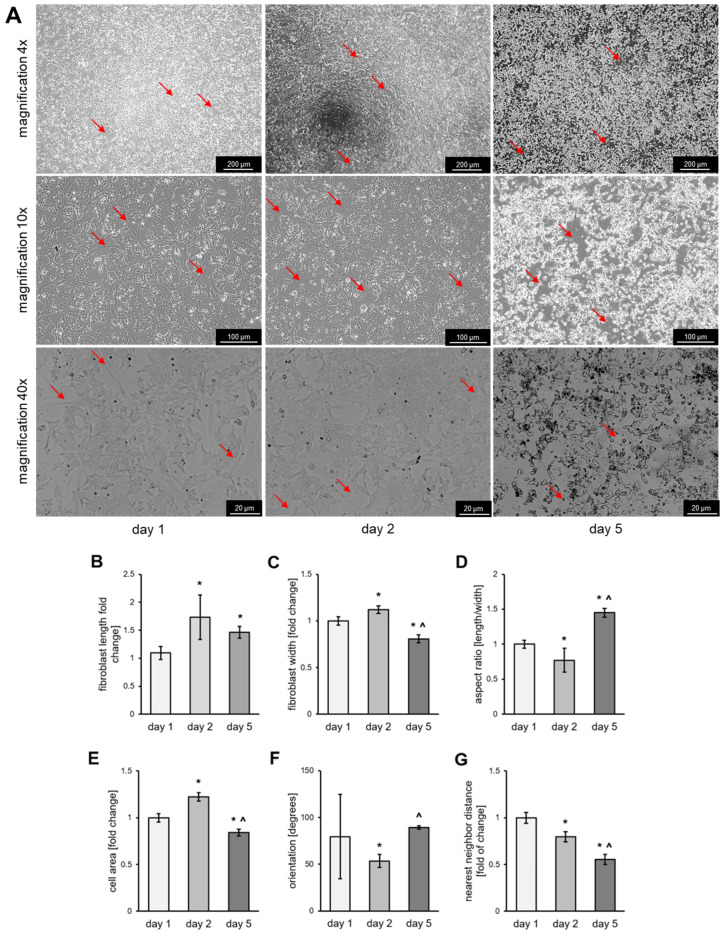
Effects of co-culture on prostate cancer cell and fibroblast morphology and spatial organization. (**A**) Representative micrographs of co-cultures of 22Rv1 prostate cancer cells and human dermal fibroblasts (HDFa) at different time points and magnifications; fibroblast-like cells are indicated by red arrows. (**B**–**G**) Quantitative morphometric analysis of fibroblast-like cells, including (**B**) cell length, (**C**) cell width, (**D**) aspect ratio (L/W), (**E**) cell area, (**F**) cell orientation (degrees), and (**G**) nearest neighbor distance (NND). Data are expressed as mean ± SD from three independent experiments. Statistical significance (*p* < 0.05): (*) vs. day 1; (^) vs. day 2.

Morphological analysis revealed differences in fibroblast-like cell morphology under co-culture conditions (Figure 5). Elongated, spindle-shaped cells were observed across all conditions but differed in abundance and organization. Increased elongation and partial alignment were noted in selected conditions, accompanied by changes in cell proportions and increased density of epithelial-like cells (Figure 5A). Quantitative morphometric analysis demonstrated changes in fibroblast morphology (Figure 5B–G), including increased cell length (Figure 5B) and aspect ratio (Figure 5D), particularly at later time points. Cell width (Figure 5C) and area (Figure 5E) increased at intermediate time points (day 2) and decreased thereafter (day 5). Orientation analysis indicated enhanced alignment (Figure 5F), while NND analysis showed reduced intercellular spacing, suggesting altered spatial organization (Figure 5G).

### 3.6. Effects of Maca Extracts on Viability of HDFa Fibroblasts

**Figure 6:**
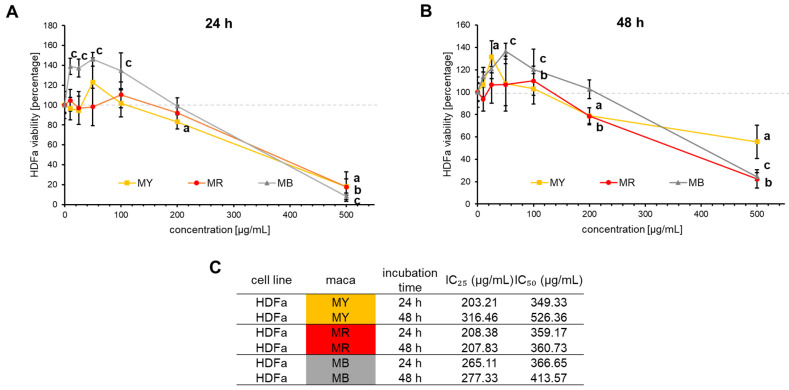
Effects of Maca Yellow (MY), Maca Red (MR), and Maca Black (MB) on the viability of human dermal fibroblasts (HDFa). Cells were treated with increasing concentrations of MY, MR, or MB for 24 h (**A**) or 48 h (**B**). (**C**) IC_25_ and IC_50_ values calculated for maca extracts in HDFa cells. Cell viability was assessed using the CellTiter-Blue assay. Inhibitory concentrations were determined from dose–response curves after 24 and 48 h of treatment. Data are expressed as mean ± SD from three independent experiments, each performed in quadruplicate (*n* = 4). The dashed line represents the viability level of the control group (100%). Statistical significance (*p* < 0.05): (a) vs. MY (0 µg/mL); (b) vs. MR (0 µg/mL); (c) vs. MB (0 µg/mL).

The effects of maca extracts on HDFa cell viability were assessed after 24 and 48 h (Figure 6A,B). MB increased viability at lower concentrations at both time points. In contrast, MY reduced viability at 200 µg/mL after 24 h, while both MY and MR decreased viability after 48 h. At the highest concentration (500 µg/mL), all extracts significantly reduced cell viability at both time points. IC_25_ and IC_50_ values indicated that MY showed the strongest cytotoxic effect at 24 h, whereas MR exhibited the greatest effect at 48 h (Figure 6C).

Thus, maca extracts affected HDFa viability in a concentration- and time-dependent manner.

### 3.7. Maca Extracts Modulate DOX-Induced Cytotoxicity in 22Rv1-HDFa Co-Cultures

**Figure 7:**
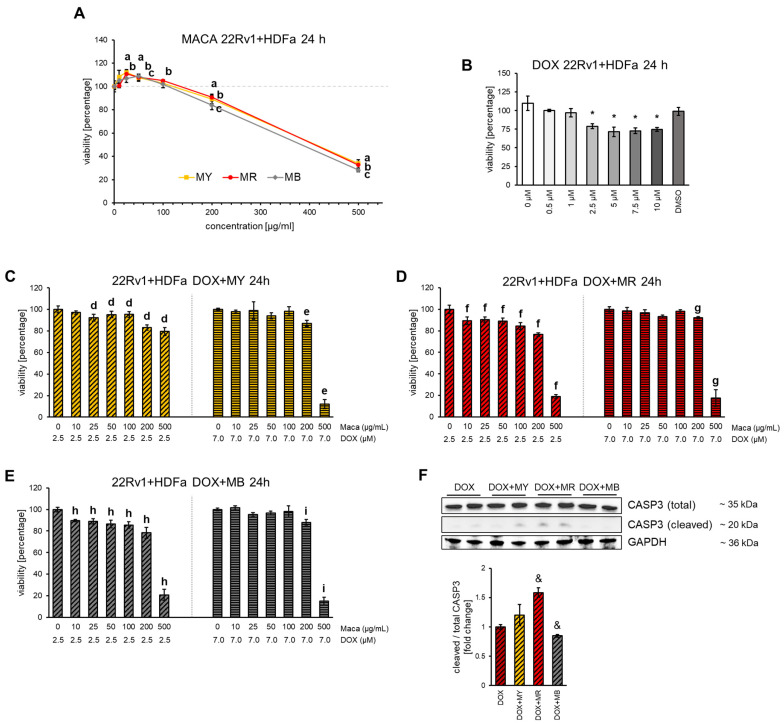
(**A**) Effects of maca extracts, Maca Yellow (MY), Maca Red (MR), and Maca Black (MB), on the viability of 22Rv1 and HDFa co-cultures. Cells were treated with increasing concentrations of maca extracts for 24 h. The dashed line represents the viability level of the control group (100%). (**B**) Effects of doxorubicin (DOX) on co-culture viability. Cells were treated with increasing concentrations of DOX for 24 h; DMSO was used as a vehicle control at a concentration equivalent to the highest DOX dose. (**C**–**E**) Effects of co-treatment with DOX and maca extracts on co-culture viability. Co-cultures were treated with DOX (2.5 or 7 µM) in combination with increasing concentrations of (**C**) MY, (**D**) MR, or (**E**) MB. (**F**) Effects of DOX (2.5 µM) and maca extracts (100 µg/mL) on caspase-3 (CASP3) levels after 24 h treatment. Protein levels of cleaved and full-length CASP3 were analyzed by Western blotting. Representative immunoblots and densitometric analyses are shown. Protein levels were normalized to GAPDH. Full-length uncropped Western blots together with membrane sectioning schemes and loading/transfer controls are provided in the Supplementary Materials. Data are presented as mean ± SD from three independent experiments. Statistical significance (*p* < 0.05): (*) vs. DOX (0 µM); (a) vs. MY (0 µg/mL); (b) vs. MR (0 µg/mL); (c) vs. MB (0 µg/mL); (d) vs. MY (0 µg/mL) + DOX (2.5 µM); (e) vs. MY (0 µg/mL) + DOX (7 µM); (f) vs. MR (0 µg/mL) + DOX (2.5 µM); (g) vs. MR (0 µg/mL) + DOX (7 µM); (h) vs. MB (0 µg/mL) + DOX (2.5 µM); (i) vs. MB (0 µg/mL) + DOX (7 µM); (&) vs. DOX-treated (2.5 µM) co-cultures.

The effects of maca extracts on co-culture viability were assessed after 24 h (Figure 7A). All extracts increased viability at lower concentrations (25–50 µg/mL) and decreased it at higher concentrations (200–500 µg/mL). The IC_25_ value for MB was higher than in 22Rv1 mono-culture and similar to HDFa alone (Table 1).

Compared with mono-culture, co-culture with HDFa fibroblasts altered responses to DOX and maca treatment. Reduced sensitivity to DOX was observed in co-culture conditions (Figure 7B), with IC_25_ (2.5 µM) and IC_50_ (7.0 µM) values increased approximately 6-fold and 3-fold, respectively, compared to mono-culture (Table 1).

Co-treatment with DOX and maca extracts further influenced viability (Figure 7C–E). At 2.5 µM DOX, all extracts reduced viability at lower concentrations, whereas at 7.0 µM DOX, this effect was mainly observed at higher maca concentrations.

The effect on apoptosis was evaluated by analyzing CASP3 levels following co-treatment with 2.5 µM DOX and 100 µg/mL maca for 24 h (Figure 7F). MR increased CASP3 levels, whereas MB reduced it.

Importantly, responses to DOX and maca co-treatment differed substantially between mono- and co-culture conditions.

### 3.8. Maca Extracts Alter Signaling-Related Protein Levels in DOX-Treated 22Rv1-HDFa Co-Cultures

**Figure 8:**
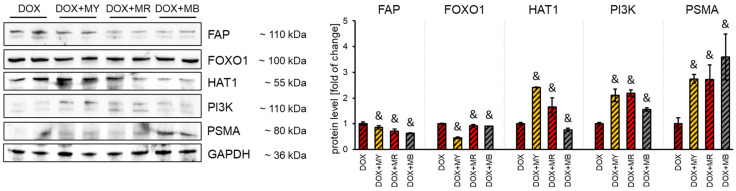
Effects of doxorubicin (DOX) and maca extracts on selected signaling-related proteins in 22Rv1-HDFa co-cultures. Protein levels of fibroblast activation protein (FAP), forkhead box protein O1 (FOXO1), histone acetyltransferase 1 (HAT1), phosphoinositide 3-kinase (PI3K), and prostate-specific membrane antigen (PSMA) were analyzed by Western blotting after 24 h treatment with DOX (2.5 µM) in combination with 100 µg/mL Maca Yellow (MY), Maca Red (MR), or Maca Black (MB). Representative immunoblots and corresponding densitometric analyses are shown. Protein levels were normalized to GAPDH. Full-length uncropped Western blots together with membrane sectioning schemes and loading/transfer controls are provided in the Supplementary Materials. Data are expressed as mean ± SD from three independent experiments. Statistical significance (*p* < 0.05): (&) vs. DOX-treated cells.

To evaluate the effect of co-treatment with maca (100 µg/mL) and DOX (2.5 µM) on selected signaling-related proteins, protein levels of FAP, FOXO1, HAT1, PI3K, and PSMA were analyzed in 22Rv1-HDFa co-cultures (Figure 8).

All extracts reduced FAP levels, most prominently MB, and decreased FOXO1, particularly in MY-treated cells. In contrast, PI3K and PSMA were consistently increased across all conditions (Figure 8). HAT1 levels were elevated by MY and MR, whereas MB reduced its expression (Figure 8).

These results indicate that maca extracts altered selected signaling markers, with distinct morphotype-dependent differences in HAT1, FAP, and FOXO1 levels.

### 3.9. Maca Extracts Decrease Lipid Droplets Accumulation and Increase ATGL in DOX-Treated 22Rv1-HDFa Co-Cultures

**Figure 9:**
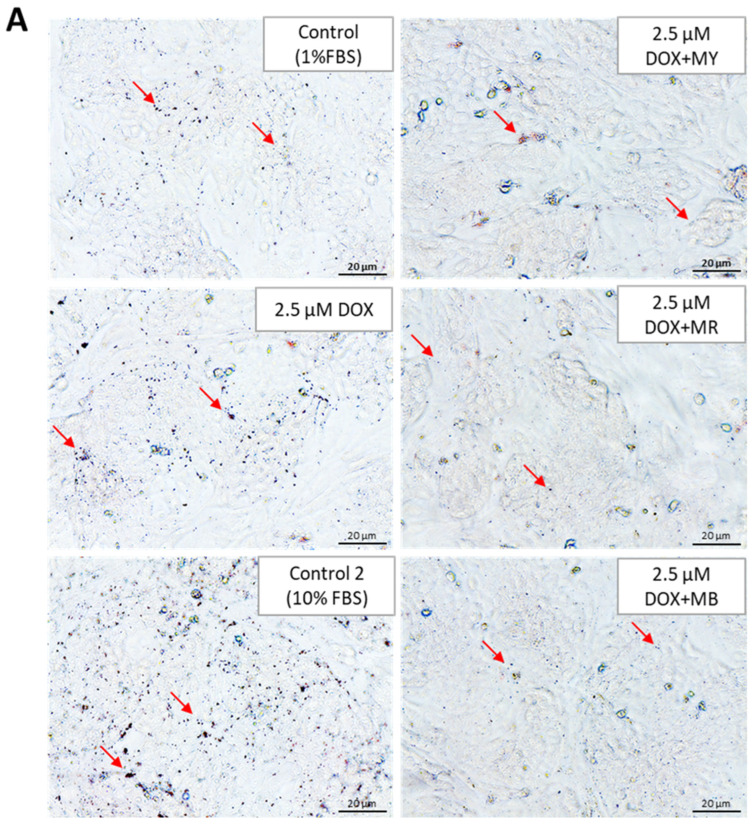

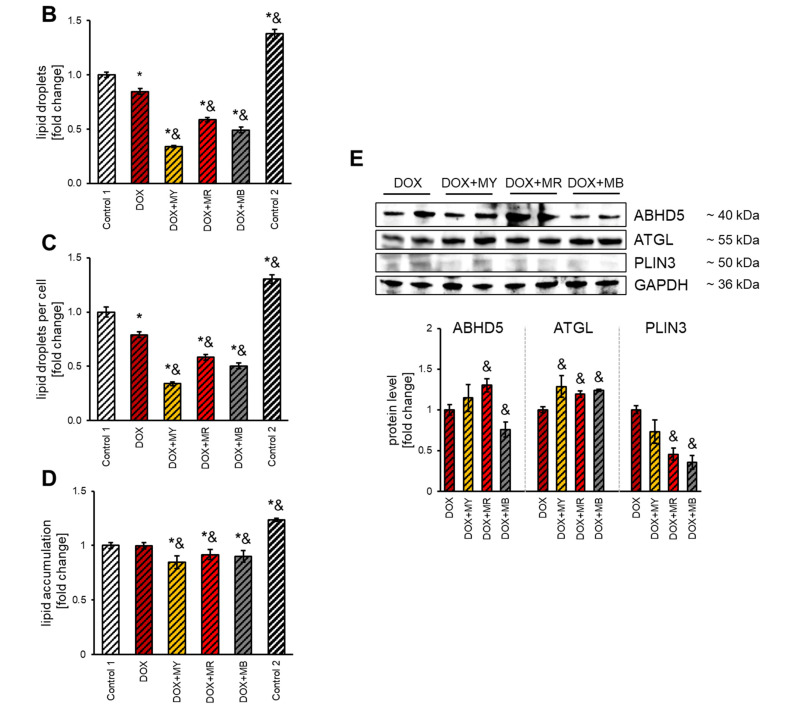
Effects of doxorubicin (DOX) and maca extracts on lipid accumulation and lipid metabolism in co-cultures of 22Rv1 prostate cancer cells and human dermal fibroblasts (HDFa). (**A**) Representative micrographs of co-cultures treated with DOX (2.5 µM) alone or in combination with 100 µg/mL Maca Yellow (MY), Maca Red (MR), or Maca Black (MB), assessed by Oil Red O (ORO) staining; lipid droplets (LDs) are indicated by red arrows. (**B**–**D**) Quantification of lipid accumulation was performed as (**B**) LDs per field of view, (**C**) LDs per cell, and (**D**) intracellular lipid content measured by ORO extraction. (**E**) Protein levels of α/β-hydrolase domain-containing protein 5 (ABHD5), adipose triglyceride lipase (ATGL), and perilipin 3 (PLIN3) were analyzed by Western blotting in co-cultures treated with DOX (2.5 µM) in combination with maca extracts (100 µg/mL); representative immunoblots and densitometric analyses normalized to the loading control are shown. Co-cultures maintained in medium containing 1% fetal bovine serum (FBS) without DOX or maca extracts served as the control (Control 1), while co-cultures cultured in full medium (2% FBS; Control 2) were used as a positive control for lipid accumulation. Representative immunoblots and corresponding densitometric analyses are shown. Protein levels were normalized to GAPDH. Full-length uncropped Western blots together with membrane sectioning schemes and loading/transfer controls are provided in the Supplementary Materials. Data are expressed as mean ± SD from three independent experiments. Statistical significance (*p* < 0.05): (*) vs. Control 1; (&) vs. DOX-treated cells.

The impact of maca (100 µg/mL) and DOX (2.5 µM) on lipid metabolism was assessed in co-cultures. ORO staining revealed reduced LDs accumulation in all maca-treated conditions (Figure 9A–D). Quantitative and semi-quantitative analyses demonstrated that maca extracts reduced lipid droplet number per field of view (Figure 9B) and per cell (Figure 9C), as well as overall intracellular lipid accumulation (Figure 9D), with the strongest effect observed for MY compared to both controls and DOX alone.

Analysis of lipolysis-related proteins showed increased ATGL levels in all conditions. The ATGL activator ABHD5 was increased in MR-treated cells and decreased in MB-treated cells, while PLIN3, a protein involved in LDs storage and turnover, was reduced in MR and MB (Figure 9E).

Taken together, maca extracts decreased LDs accumulation in DOX-treated co-cultures, which was accompanied by altered levels of lipid metabolism-related proteins of ATGL, ABHD5 and PLIN3.

## 4. Discussion

Comparison of mono-culture and co-culture models demonstrated that stromal interactions substantially altered cellular responses to DOX and maca treatment, particularly signaling and lipid metabolism markers. While mono-culture systems reflect cell-intrinsic responses, they do not capture the complexity of tumor–stroma interactions involved in cancer progression, metabolism, and therapy response [3]. In contrast, co-culture with fibroblasts provides a simplified but biologically relevant model of tumor–stroma interactions that can significantly influence cellular behavior [14,15].

One key observation was that cytotoxic responses differed markedly between models. HDFa fibroblasts were less sensitive to DOX than 22Rv1 prostate cancer cells, and co-culture conditions required substantially higher DOX concentrations to achieve effects comparable to mono-culture. These findings are consistent with previous reports linking CAF-associated stromal interactions to therapy resistance through extracellular matrix remodeling, paracrine signaling, and metabolic support [11,14,20]. Fibroblasts may additionally support cancer cell survival by influencing lipid metabolism and cellular stress responses under co-culture conditions [14,15,18,20], which may explain the increased IC values and altered apoptotic responses observed in co-culture.

Morphological and morphometric analyses further revealed significant morphological changes in fibroblast-like cells in co-culture. Proliferation-related effects were evaluated using a wound healing assay conducted under reduced-serum conditions (1% FBS), which substantially limits migratory activity and shifts wound closure dynamics toward proliferation-driven processes. Nevertheless, it is important to recognize that wound healing assays cannot fully distinguish between cell proliferation and residual migration. Therefore, the observed changes should be interpreted as reflecting combined proliferation-associated responses rather than a direct and exclusive measure of cell proliferation. Further studies employing proliferation-specific assays, such as EdU incorporation or cell cycle analysis, would be required to confirm these findings. However, increased elongation, higher aspect ratio, reduced cell width, and enhanced alignment may reflect cytoskeletal reorganization and altered fibroblast-like morphology under co-culture conditions [13,20]. Additionally, decreased NND and changes in spatial distribution may reflect altered spatial organization and cell distribution within the culture [20]. The observed remodeling of fibroblast-like cells may reflect stromal adaptation under co-culture conditions. Although HDFa were used to model tumor–stroma interactions, it should be noted that these cells do not fully recapitulate the phenotype of CAFs. The observed morphological changes suggest partial fibroblast activation; however, in the absence of specific CAF markers and functional validation, these findings should be interpreted as indicative of a fibroblast activation-like state rather than a true CAF phenotype. As these conclusions are based primarily on morphological observations without molecular or functional validation of CAF differentiation, they should be interpreted with caution, as the observed changes may also reflect differences in proliferation, survival, or spatial competition rather than definitive evidence of CAF activation.

### 4.1. Context-Dependent Modulation of Apoptosis

Consistent with previous reports, maca can influence apoptosis in a context-dependent manner, including both decreases and increases in CASP3 levels depending on experimental conditions [27,28,29]. In our study, MR decreased both CASP3 and CASP8 levels in 22Rv1 mono-culture, indicating attenuation of apoptotic signaling. In contrast, MB reduced CASP3 but increased CASP8, suggesting partial uncoupling of apoptotic pathways, where initiator caspase signaling was not accompanied by strong downstream apoptotic responses. This may reflect a non-apoptotic or stress-related role of CASP8, which has been reported to participate in alternative signaling pathways beyond apoptosis [30]. Importantly, this pattern was altered in co-culture with HDFa fibroblasts, highlighting the influence of stromal interactions under co-culture conditions. While MB maintained reduced CASP3 levels, MR led to increased CASP3, indicating that stromal interactions altered apoptosis marker responses in a morphotype-dependent manner. Such divergence is consistent with the known plasticity of tumor–stroma interactions, where fibroblasts can either suppress apoptosis and promote survival or, under certain conditions, enhance apoptotic signaling through modulation of death receptor pathways [15]. Collectively, our findings highlight the importance of incorporating stromal interaction models into in vitro studies.

### 4.2. Phenotype-Dependent Changes in Signaling-Related Proteins Under Co-Culture Conditions

Distinct morphotype-dependent effects of maca were observed. All extracts increased PSMA levels in 22Rv1 mono-culture and PSMA together with PI3K in co-culture, whereas FOXO1, FAP, and HAT1 showed differential regulation. These differences are consistent with variability in phytochemical composition among maca phenotypes. MB is enriched in macamides and macaenes and shows stronger effects on proliferation and apoptosis pathways, MR contains higher levels of glucosinolates and polyphenols, and MY exhibits a comparatively balanced metabolite profile [21,22,23].

Available evidence suggests that maca-derived phytochemicals may influence signaling proteins involved in prostate cancer progression, including the PI3K/AKT/FOXO axis [8,26]. This is further supported by reports indicating that maca bioactive compounds, including macamides and glucosinolates, exhibit biological activities associated with modulation of cell proliferation [21,22,25]. Consistently, the concurrent increase in PI3K and decrease in FOXO1 observed in co-cultures may reflect altered PI3K/FOXO1 signaling rather than direct evidence of pathway activation. Since FOXO1 functions as a tumor suppressor negatively regulated by PI3K/AKT signaling [4], its downregulation may indicate altered survival signaling under co-culture conditions.

In contrast, no direct evidence links maca extracts to the regulation of HAT1, PSMA, or FAP. PSMA, a marker linked to prostate cancer progression [5,6,7], was consistently increased, potentially reflecting stromal interaction-dependent effects [14,20]. In contrast, reduced FAP levels may indicate partial attenuation of fibroblast activation-like features under co-culture conditions [11,13]. Although no direct evidence links maca extracts to HAT1 regulation, the differential HAT1 expression observed between morphotypes, supports morphotype-specific biological activity potentially associated with phytochemical variability [22], may reflect indirect effects associated with chromatin-related processes [10].

Under DOX treatment, reduced sensitivity in HDFa-22Rv1 co-culture further emphasizes the importance of stromal interactions in shaping treatment responses. Taken together, these findings suggest that stromal interactions substantially influence signaling, metabolic regulation, and treatment responses under co-culture conditions.

### 4.3. Changes in Lipid Metabolism-Related Markers Following Maca Treatment

A consistent effect observed in both mono- and co-culture systems was reduced LD accumulation following maca treatment, particularly with MY. Since increased LD accumulation has been linked to prostate cancer progression and therapy resistance [16], these findings may reflect altered lipid utilization, supported by reduced LD levels together with increased ATGL expression. This effect was particularly evident under co-culture conditions and during DOX treatment, where elevated ATGL levels accompanied reduced LD accumulation and reduced sensitivity to DOX.

In mono-culture, ATGL elevation was observed predominantly with MB, suggesting morphotype-specific metabolic effects. Although no direct evidence links maca extracts to ATGL regulation, MB has been reported to modulate peroxisome proliferator-activated receptor alpha (PPARα) signaling and fatty acid oxidation in metabolic models [31], which may partially explain the observed changes. Similarly, ABHD5, a key co-activator of ATGL [18], showed morphotype-dependent regulation, with increased levels in MR, decreased levels in MB, and no change in MY. This finding suggests that MR may enhance ATGL- and ABHD5-related lipid remodeling, whereas MB may promote lipolysis through alternative mechanisms potentially linked to PPARα signaling [31].

The concomitant increase in ATGL and PI3K, together with decreased FOXO1, may further indicate altered metabolic and stress-related signaling under co-culture conditions [9]. In addition, decreased PLIN3, a LD-associated protein involved in LDs formation and protection against lipolysis [19], was consistent with the observed reduction in LD accumulation. Altogether, these findings suggest that maca extracts modulate markers linked to fatty acid oxidation and PI3K/FOXO1 signaling [26], particularly under co-culture conditions.

Collectively, our findings suggest that stromal interactions substantially influence cellular responses to DOX and maca treatment. Compared with mono-culture, co-culture altered cytotoxic, signaling, and lipid metabolism responses that were not observed under mono-culture conditions. These observations support the idea that maca extracts modulate cancer cell behavior in a context-dependent manner, with stromal interactions strongly shaping the observed effects.

### 4.4. Limitations and Further Studies

This study has several limitations. The co-culture model represents a simplified tumor–stroma interaction system and does not fully recapitulate the complexity of the in vivo tumor microenvironment. Fibroblast activation was assessed indirectly using morphology and FAP expression without broader CAF marker or cytokine analyses. In addition, only one prostate cancer cell line and one fibroblast model were used.

Another limitation is the use of crude maca extracts without characterization of specific bioactive compounds. The use of crude extracts without phytochemical standardization limits direct attribution of the observed effects to specific bioactive compounds. Furthermore, mechanistic conclusions are limited because pathway activity was inferred primarily from protein expression changes without functional validation.

Importantly, the present findings do not support a direct chemosensitizing or therapeutic effect of maca extracts under the tested conditions. Maca did not enhance DOX-induced cytotoxicity and, in several conditions, increased cell viability. Therefore, the observed effects should be interpreted primarily as context-dependent modulation of cellular responses rather than direct anticancer activity.

Future studies should include additional prostate cancer models, cytokine profiling, advanced co-culture systems, and mechanistic validation approaches to better define the biological significance of maca-induced effects.

## 5. Conclusions

**Figure 10:**
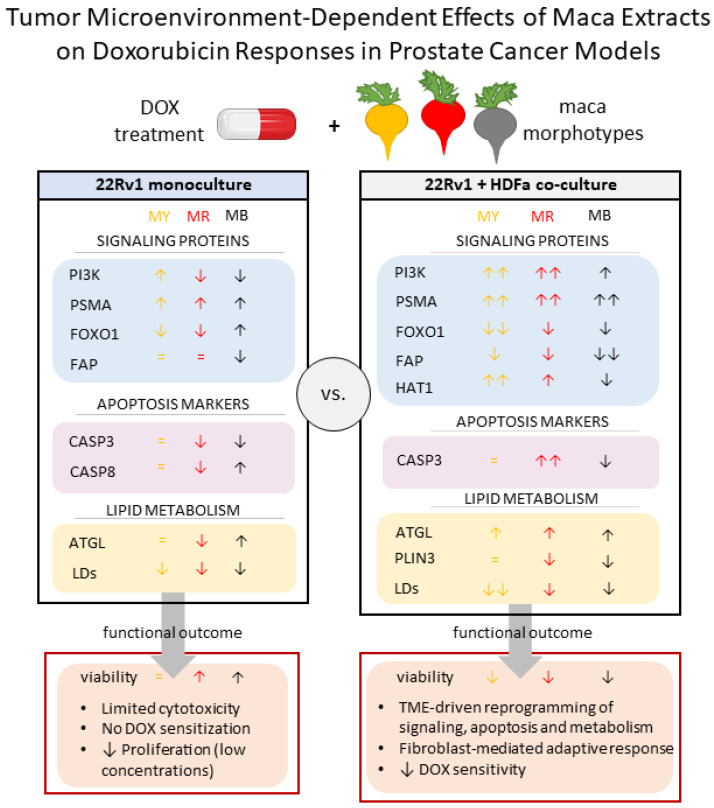
Tumor microenvironment (TME)-dependent effects of maca extracts on signaling pathways, apoptosis, and lipid metabolism in 22Rv1 prostate cancer cells treated with doxorubicin (DOX). In mono-culture conditions, maca extracts exert limited cytotoxicity, with modest modulation of signaling pathways and no enhancement of DOX sensitivity, while reducing wound closure at low concentrations. In contrast, co-culture with human dermal fibroblasts (HDFa) induces changes in signaling, apoptosis, and lipid metabolism. This is associated with a fibroblast-mediated adaptive response and a marked reduction in DOX sensitivity. Differential modulation of PI3K/PSMA/FOXO1/FAP signaling and lipid metabolism markers (ATGL, PLIN3, lipid droplets) highlights the role of stromal interactions in shaping prostate cancer cell responses to treatment. 22Rv1, human prostate cancer cell line; ATGL, adipose triglyceride lipase; CASP3, caspase-3; CASP8, caspase-8; FAP, fibroblast activation protein; FOXO1, forkhead box protein O1; HAT1, histone acetyltransferase 1; HDFa, human dermal fibroblasts; LDs, lipid droplets; MB, Maca Black; MR, Maca Red; MY, Maca Yellow; PI3K, phosphoinositide 3-kinase; PLIN3, perilipin 3; PSMA, prostate-specific membrane antigen. ↑ indicates an increase, ↓ indicates a decrease, and = indicates no change relative to the DOX control.

In conclusion, maca extracts showed limited direct cytotoxicity and did not enhance the cytotoxic effect of DOX in 22Rv1 prostate cancer cells under the tested conditions. Instead, maca treatment was associated with context- and morphotype-dependent changes in selected apoptosis-, signaling-, and lipid metabolism-related proteins. Co-culture with HDFa fibroblasts altered cellular responses compared with mono-culture, suggesting that stromal interactions may influence cellular responses to maca extracts (Figure 10). However, these findings should be interpreted as exploratory and correlative. Further studies using additional prostate cancer models, normal prostate-derived cells, cytokine profiling, defined maca fractions, and functional pathway validation are required to determine the biological relevance and mechanistic basis of these observations.

## Figures and Tables

**Table 1:** Calculated IC_25_ and IC_50_ values for doxorubicin (DOX) and maca extracts, Maca Yellow (MY), Maca Red (MR), and Maca Black (MB), in co-cultures of 22Rv1 prostate cancer cells and human dermal fibroblasts (HDFa). Inhibitory concentrations were determined from dose–response curves after 24 h of treatment. Values are expressed as means from three independent experiments (*n* = 4 per experiment).

Co-Culture	Substance	Incubation Time	IC_25_ (µg/mL)	IC_50_ (µg/mL)
22Rv1 + HDFa	DOX	24 h	3.03	6.71
22Rv1 + HDFa	MY	24 h	251.18	420.22
22Rv1 + HDFa	MR	24 h	247.35	419.64
22Rv1 + HDFa	MB	24 h	226.75	385.99

## Data Availability

The raw data supporting the findings of this study are available from the corresponding author upon reasonable request.

## Supplementary Materials

The following supporting information can be downloaded at: https://www.mdpi.com/article/10.3390/cells15121090/s1. Figure S1: DOX-induced changes in 22Rv1 cell viability and corresponding IC_25_/IC_50_ values (related to Section 3.2). Figure S2: Representative ORO staining microscopic images (corresponding to Figure 4A); Figure S3: Representative micrographs of co-cultures of 22Rv1 prostate cancer cells and human dermal fibroblasts (HDFa) (corresponding to Figure 5A); Figure S4: Representative micrographs of co-cultures ORO staining (corresponding to Figure 9A).

## Abbreviations

The following abbreviations are used in this manuscript:

ABHD5	α/β-hydrolase domain-containing protein 5
AKT	protein kinase B
ATGL	adipose triglyceride lipase
CAFs	cancer-associated fibroblasts
CASP3	caspase-3
CASP8	caspase-8
DMSO	dimethyl sulfoxide
DOX	doxorubicin
FAP	fibroblast activation protein
FBS	fetal bovine serum
FOXO1	forkhead box O1
GAPDH	glyceraldehyde-3-phosphate dehydrogenase
HAT1	histone acetyltransferase 1
HDFa	human dermal fibroblasts
LDs	lipid droplets
MB	maca black
MR	maca red
MY	maca yellow
NND	nearest neighbor distance
ORO	Oil Red O
PBS	phosphate-buffered saline
PI3K	phosphoinositide 3-kinase
PLIN3	perilipin 3
PSMA	prostate-specific membrane antigen
PPARα	peroxisome proliferator-activated receptor alpha
TME	tumor microenvironment
