# Supplementary material for: Context-Dependent Effects of Maca Extracts on Signaling, Apoptosis, and Lipid Metabolism Markers in Prostate Cancer Mono- and Co-Culture Models
Source: Cells. 2026 Jun 16;15(12):1090. doi: 10.3390/cells15121090 (PMC13297626; doi:10.3390/cells15121090)
Supplement: Supplementary file 1 [file cells-15-01090-s001.zip › cells-4307431-supplementary.pdf]

A

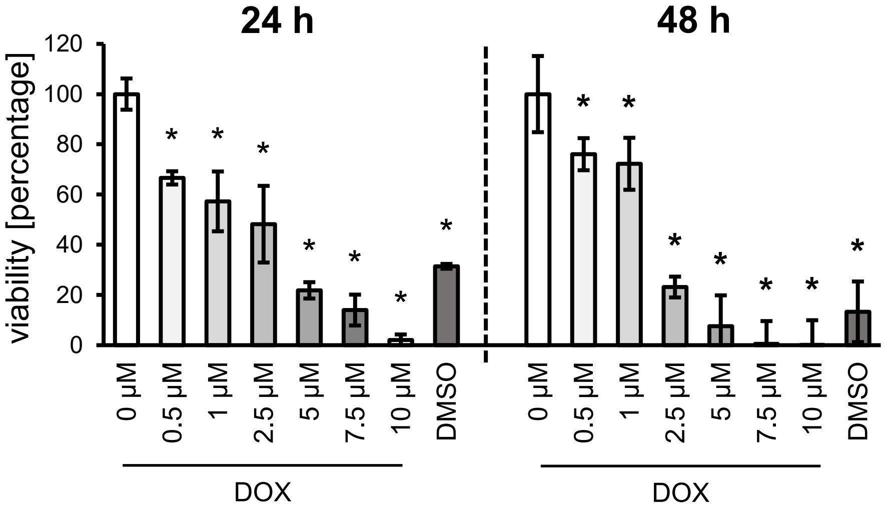

B

cell line	substance	incubation time	IC_{25} (µM)	IC_{50} (µM)
22Rv1	DOX	24 h	0.53	2.49
22Rv1	DOX	48 h	0.62	2.19

Supplementary Figure 1. (A) Effects of doxorubicin (DOX) on the viability of prostate cancer cells. 22Rv1 cells were treated with increasing concentrations of DOX for 24 or 48 h. (B) Calculated IC_{25} and IC_{50} values for DOX in 22Rv1 cells. Inhibitory concentrations were determined from dose–response curves after 24 and 48 h of treatment. Cell viability was assessed using the CellTiter-Blue assay. DMSO was used as a vehicle control at a concentration equivalent to that in the highest DOX dose. Data are expressed as mean ± SD from three independent experiments, each performed in quadruplicate (n = 4). Statistical significance ($p < 0.05$): (*) vs. DOX (0 µM).

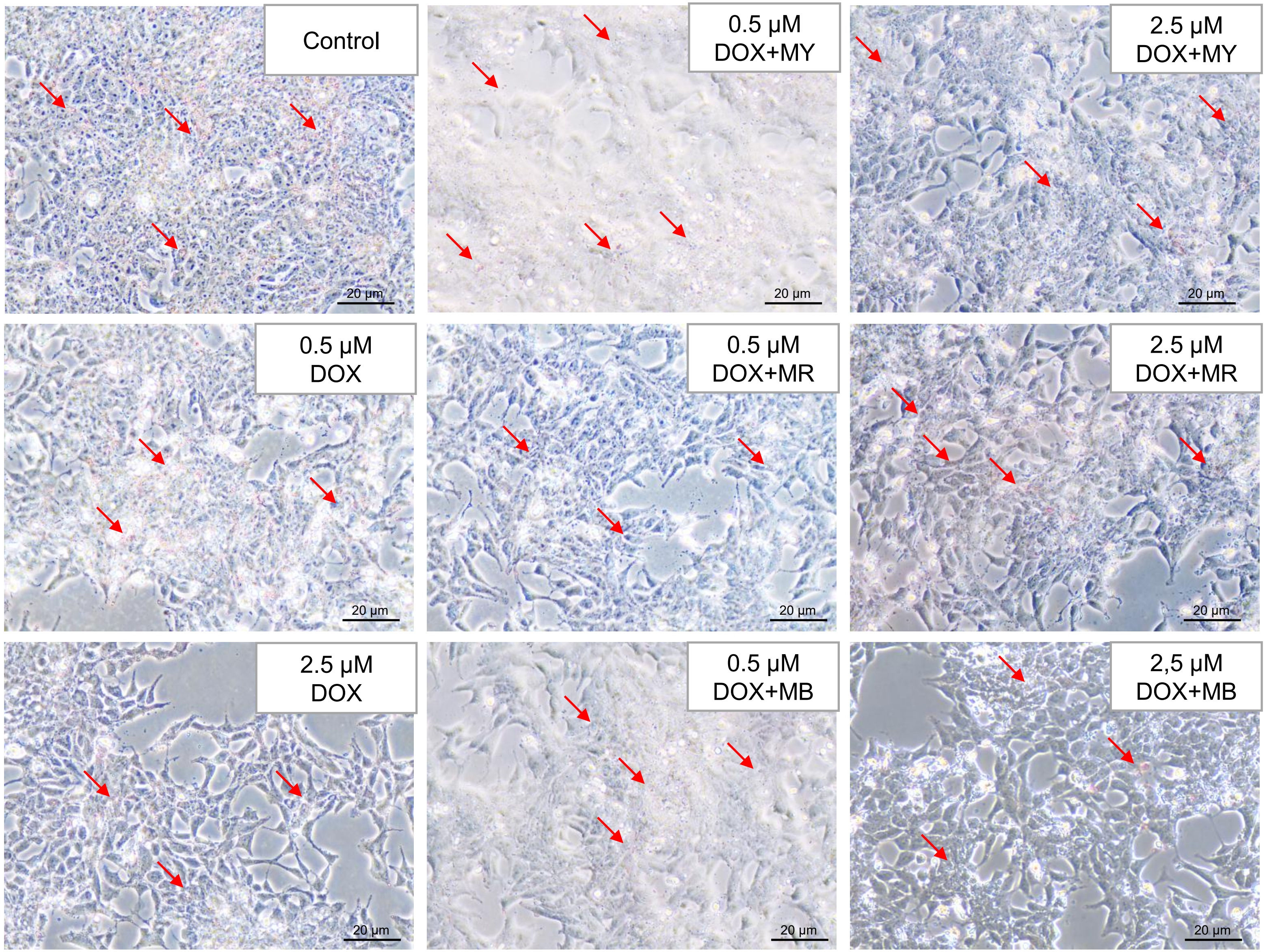

Figure S2: Representative ORO staining microscopic images (corresponding to Figure 4A).

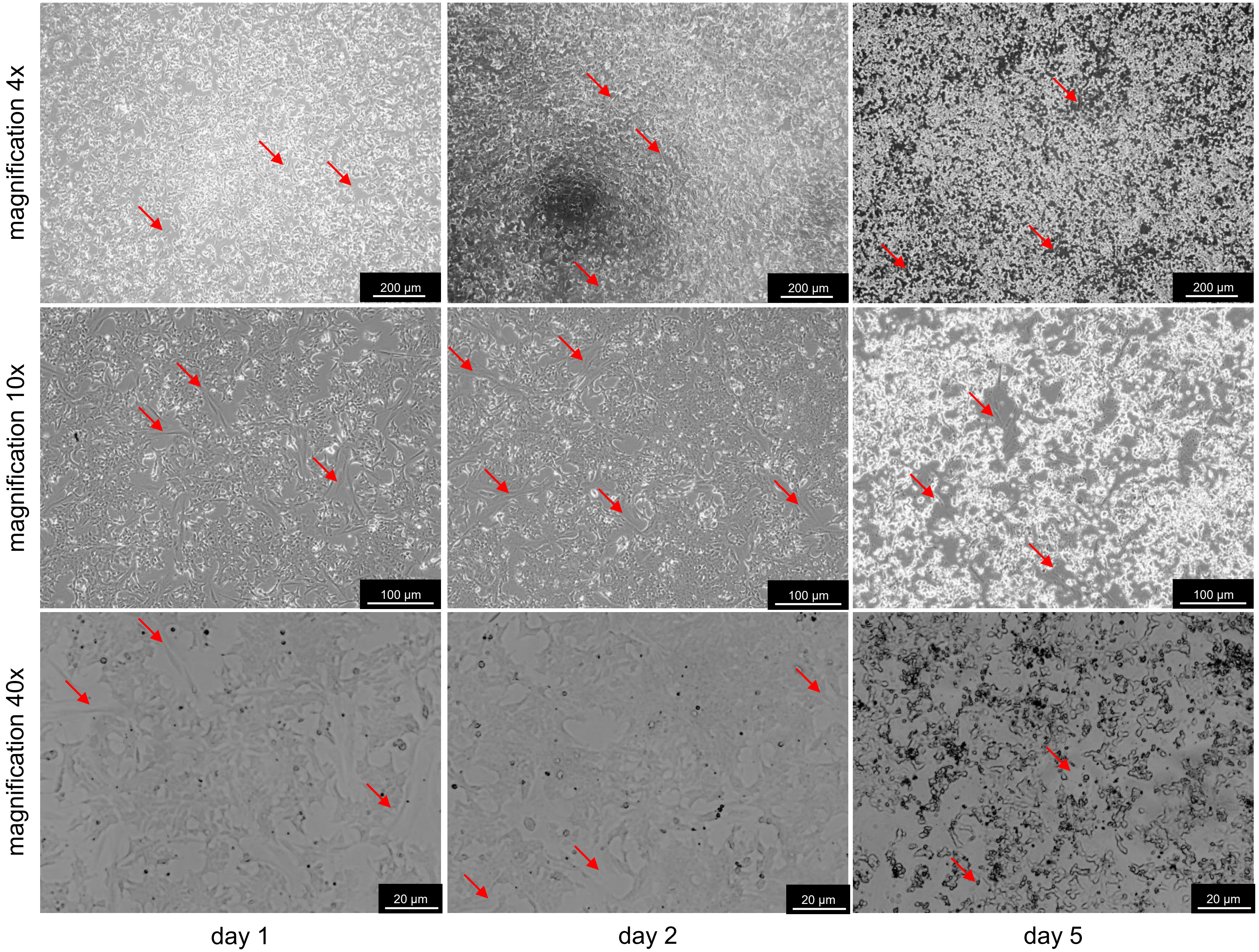

Figure S3: Representative micrographs of co-cultures of 22Rv1 prostate cancer cells and human dermal fibroblasts (HDFa) (corresponding to Figure 5A).

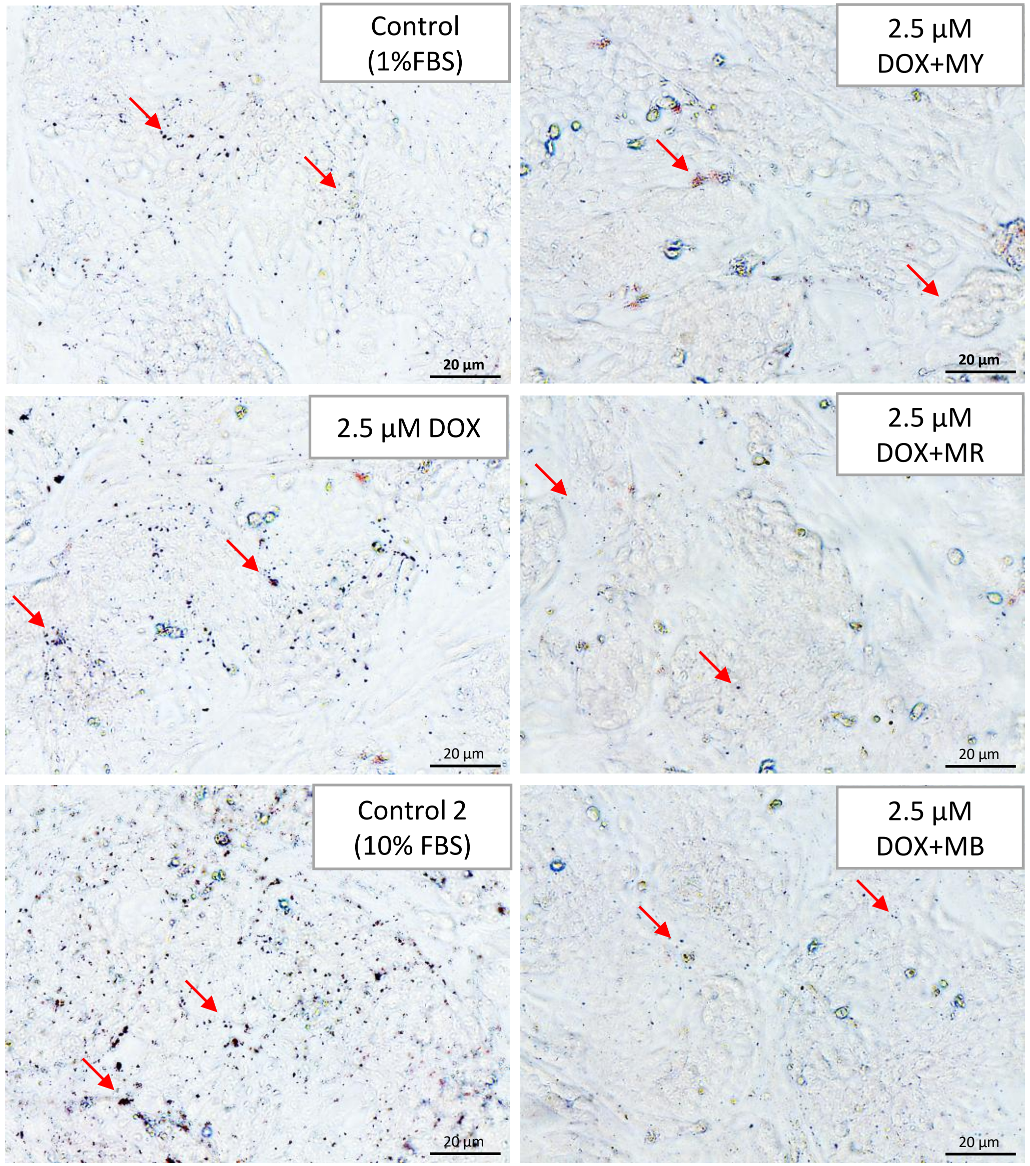

Figure S4: Representative micrographs of co-cultures ORO staining (corresponding to Figure 9A).

Full-length uncropped Western blot images are presented together with gel electrophoresis images, post-transfer gels, post-transfer membranes, and membrane sectioning schemes used for protein detection. These data document protein loading, transfer efficiency, and the relationship between individual membrane sections and the corresponding protein targets.

RAW WB 22Rv1

Abbreviations: Liver, mouse liver tissue (positive control), DOX, doxorubicin, MY, yellow maca, MR, red maca, MB, black maca, 22Rv1, human prostate cancer cell line, HDFa, adult human dermal fibroblasts; c-cul, Co-Culture.

Raw WB 22Rv1

gel electrophoresis

Gel 2

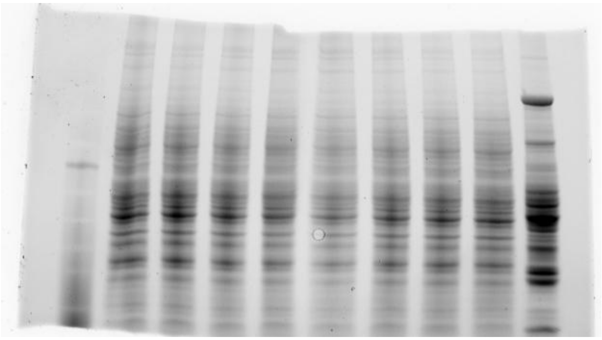

post-transfer gel

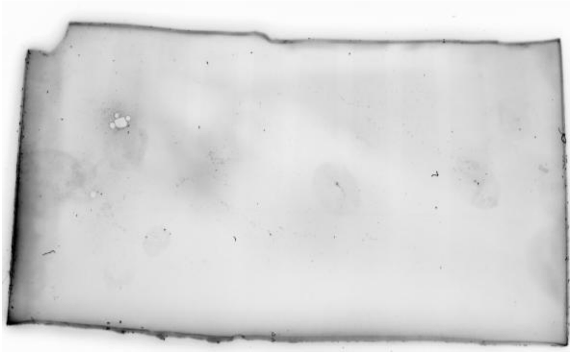

post-transfer membrane

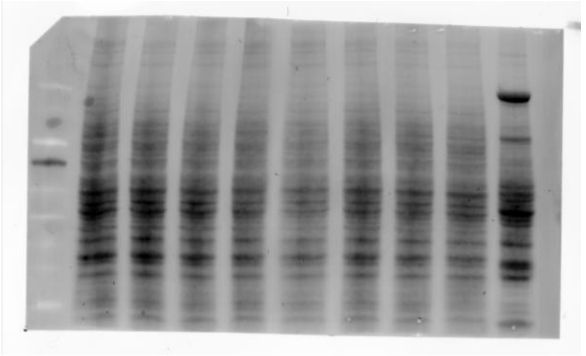

post-transfer membrane

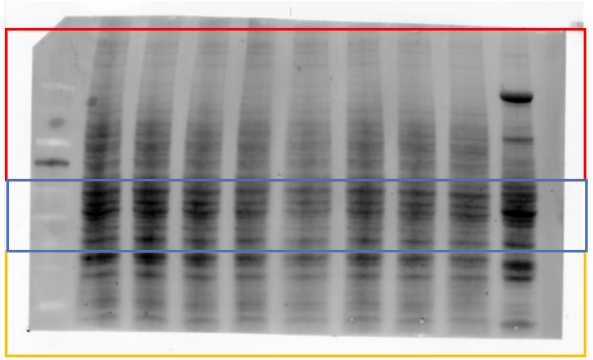

2.1

2.2

2.3

ChemiDoc image of the post-transfer membrane

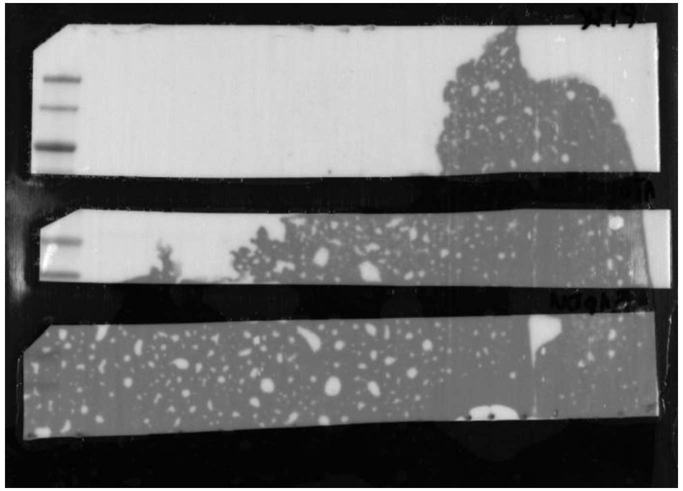

2.1

2.2

2.3

Raw WB 22Rv1

2.1 PI3K
anti-Rabbit

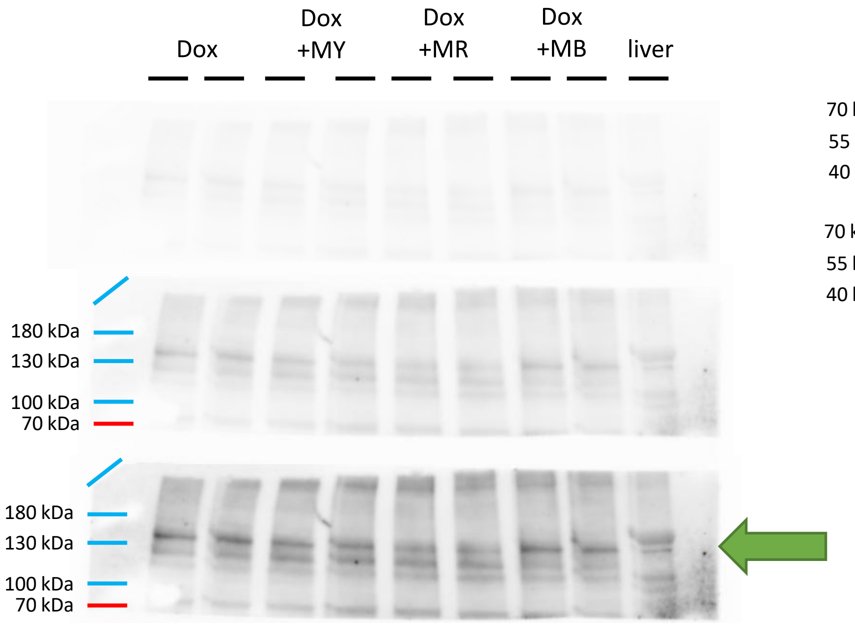

Used for Figure 3C

2.2 ATGL
anti-Rabbit

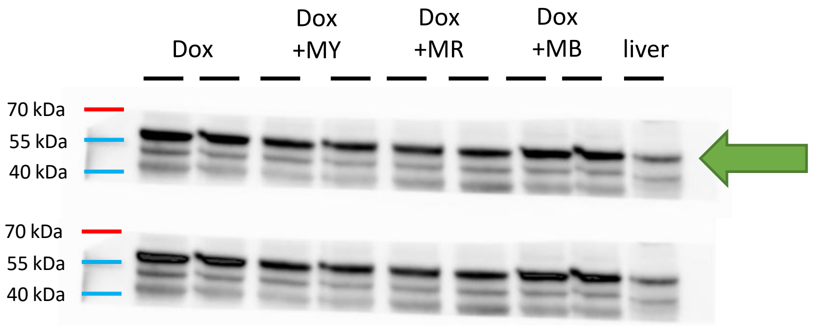

Used for Figure 4 E

2.3 GAPDH
anti-Mouse

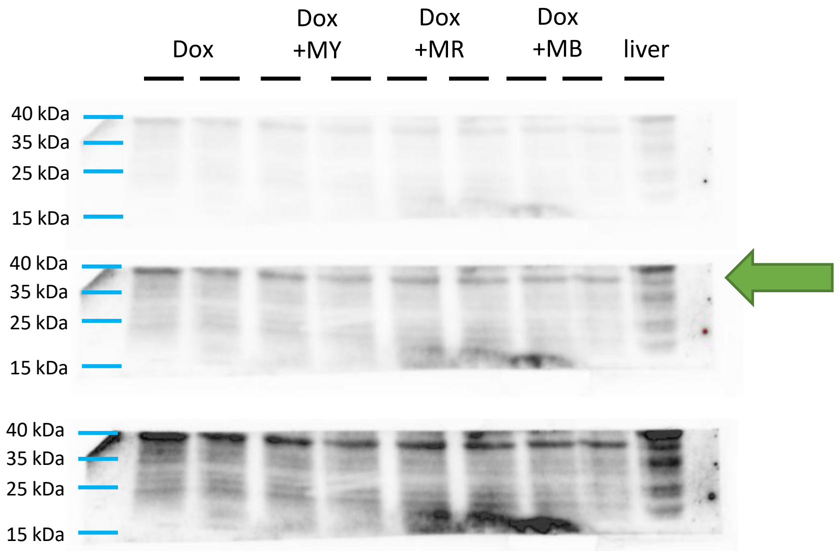

Used for Figure 3C/4E

2.1

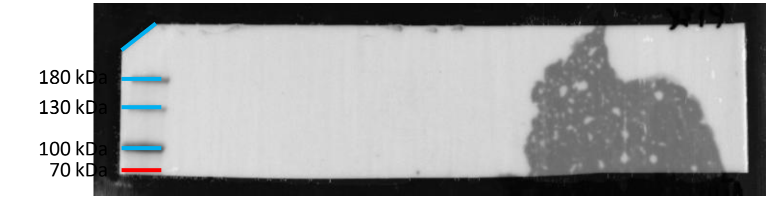

2.2

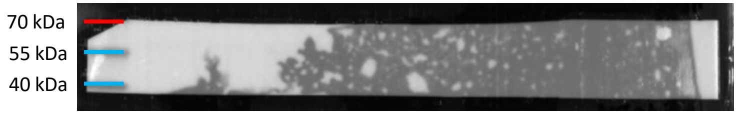

2.3

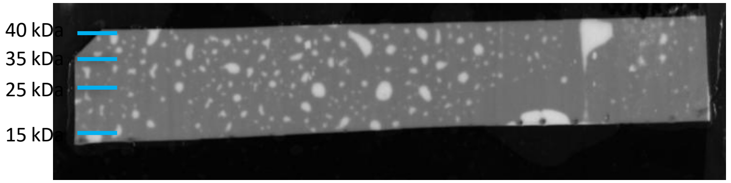

Raw WB 22Rv1

gel electrophoresis

Gel 3

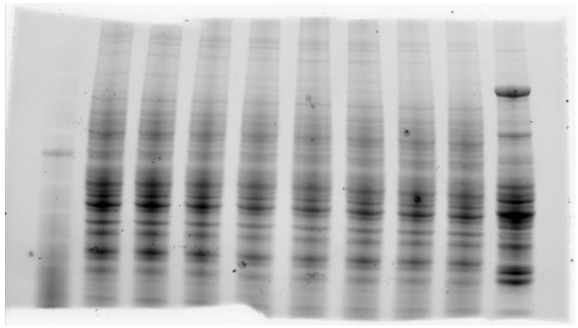

post-transfer gel

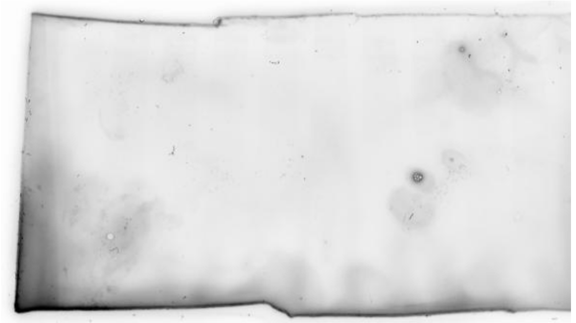

post-transfer membrane

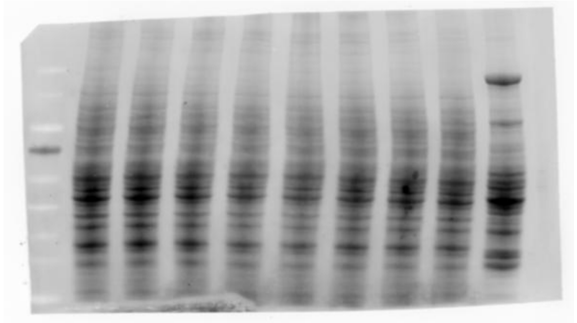

post-transfer membrane

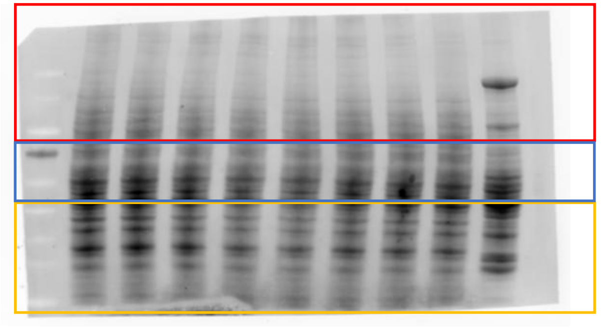

ChemiDoc image of the post-transfer membrane

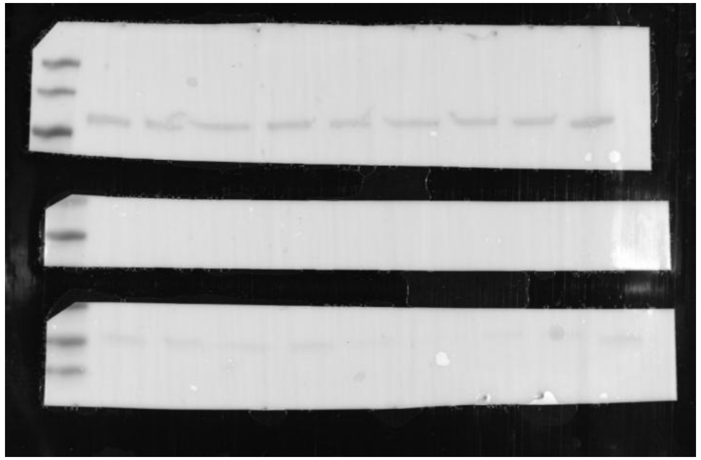

Raw WB 22Rv1

3.1 FOXO1
anti-Rabbit

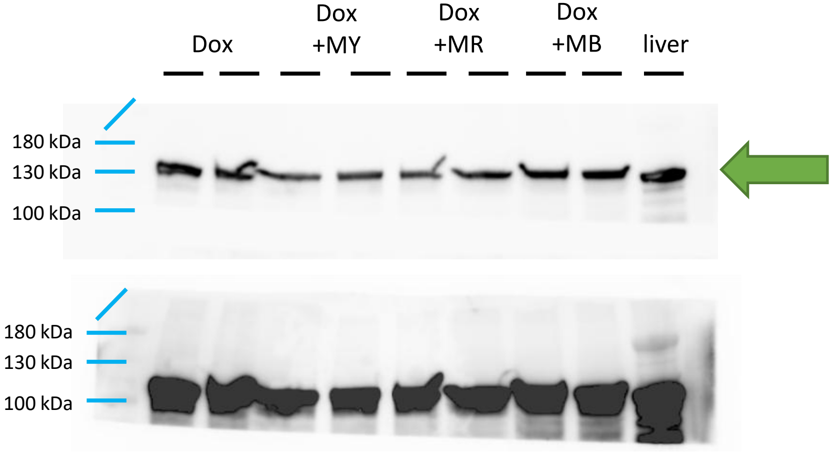

Used for Figure 3C

3.2

Not used

3.3 CASP3
anti-Rabbit

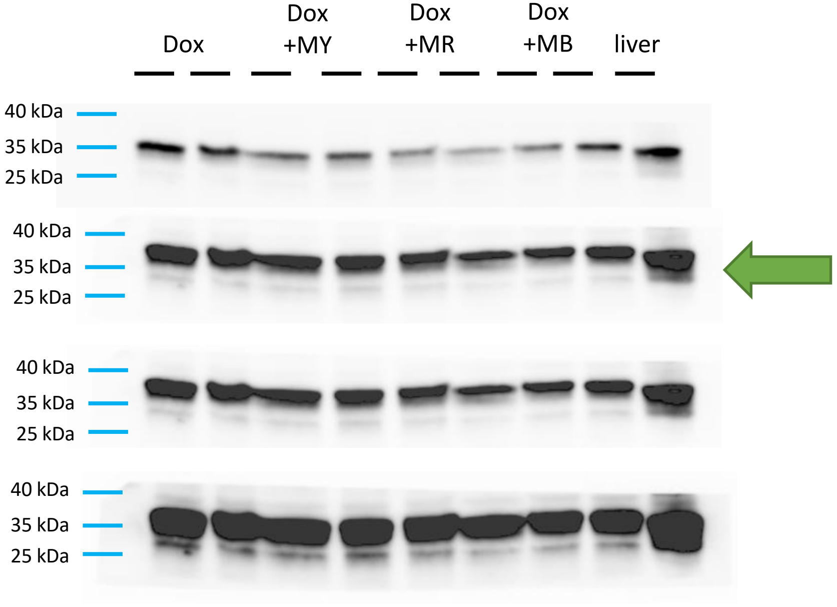

Used for Figure 2D

3.1

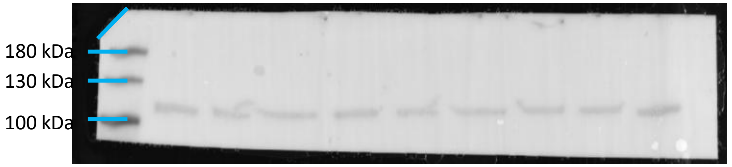

3.2

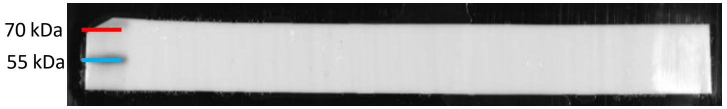

3.3

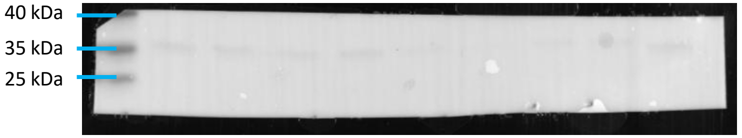

Raw WB 22Rv1

Gel 4

gel electrophoresis

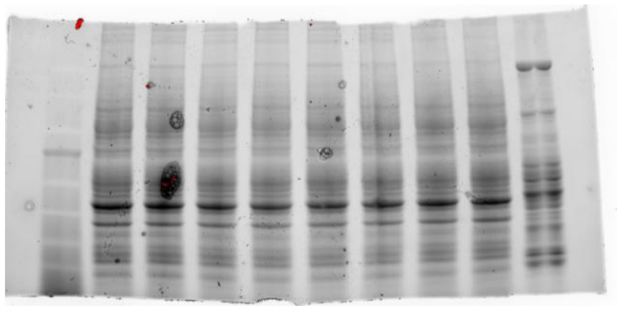

post-transfer gel

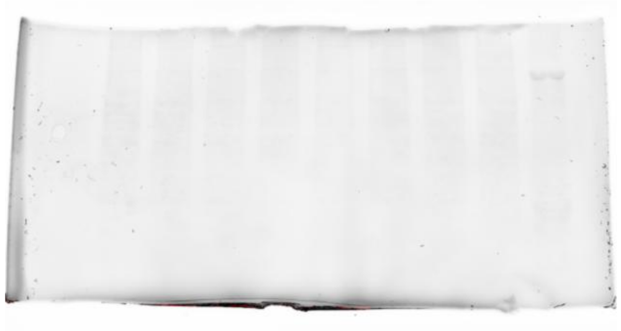

post-transfer membrane

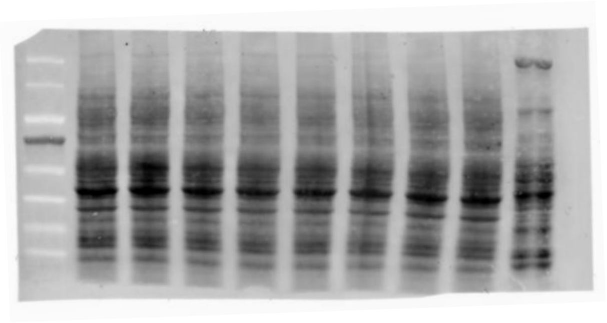

post-transfer membrane

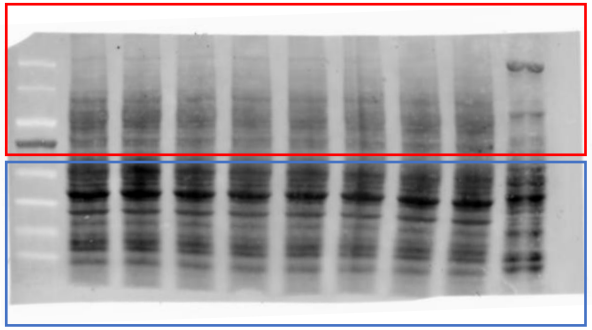

4.1

4.2

ChemiDoc image of the post-transfer membrane

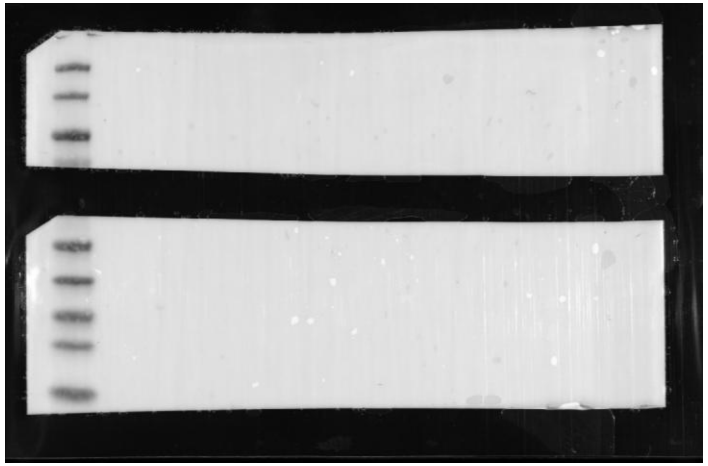

4.1

4.2

Raw WB 22Rv1

4.1

Not used

4.2 CASP8
anti-Mouse

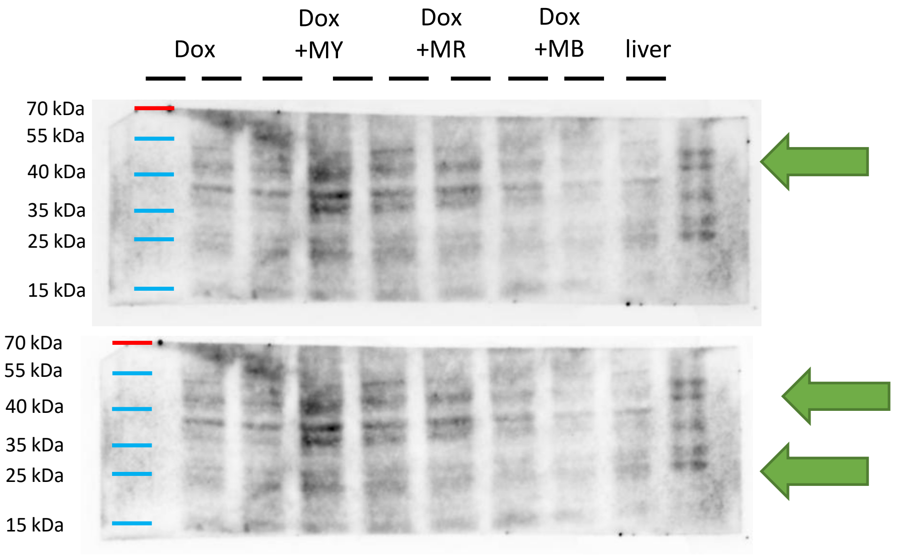

Used for Figure 2D

4.1

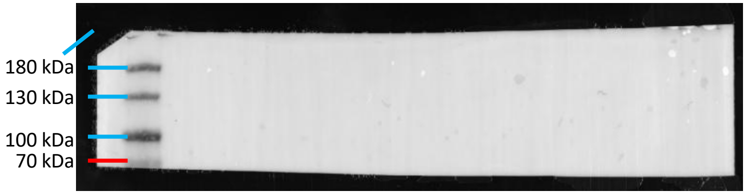

4.2

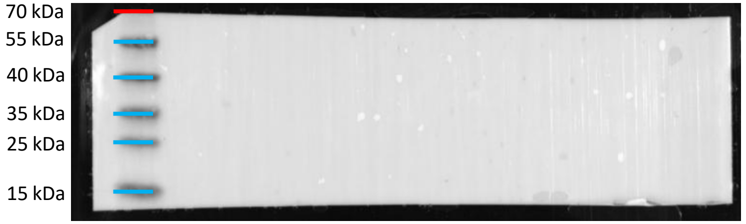

Raw WB 22Rv1

gel electrophoresis

post-transfer gel

post-transfer membrane

Gel 5

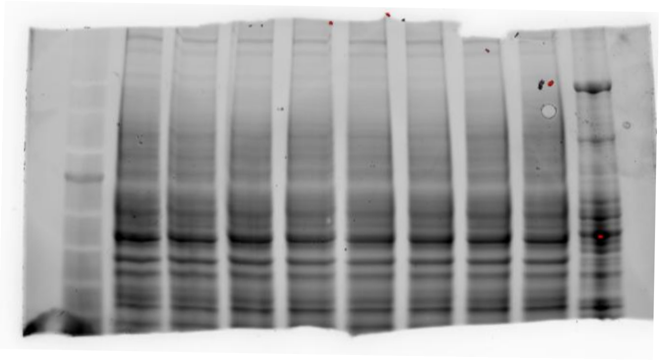

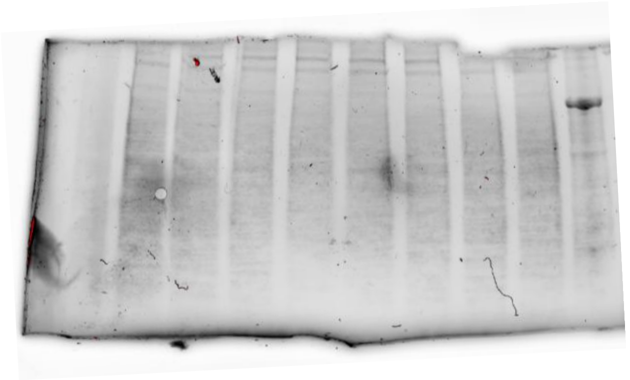

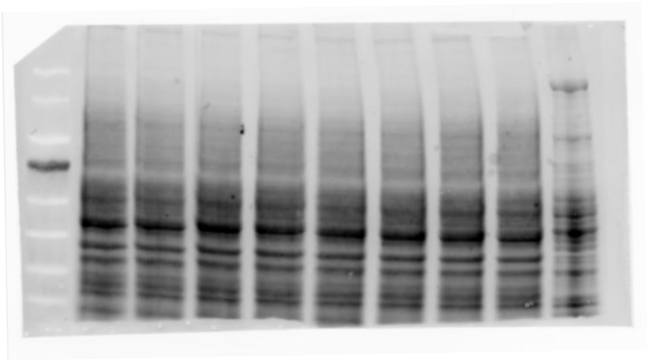

post-transfer membrane

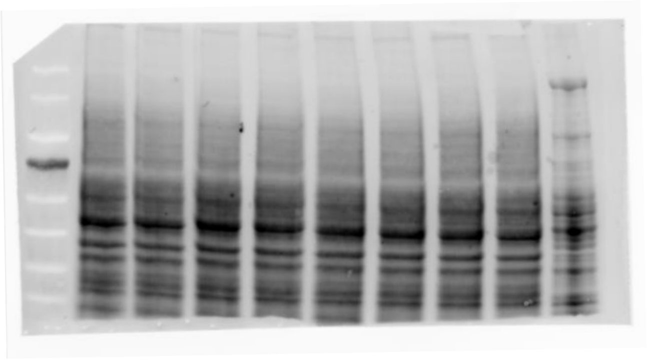

5

ChemiDoc image of the post-transfer membrane

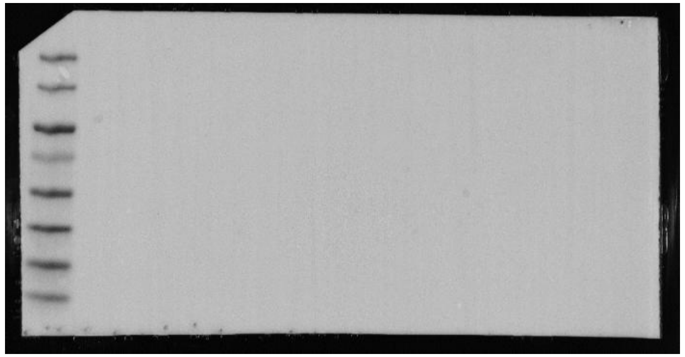

5

Raw WB 22Rv1

5 PSMA
anti-Mouse

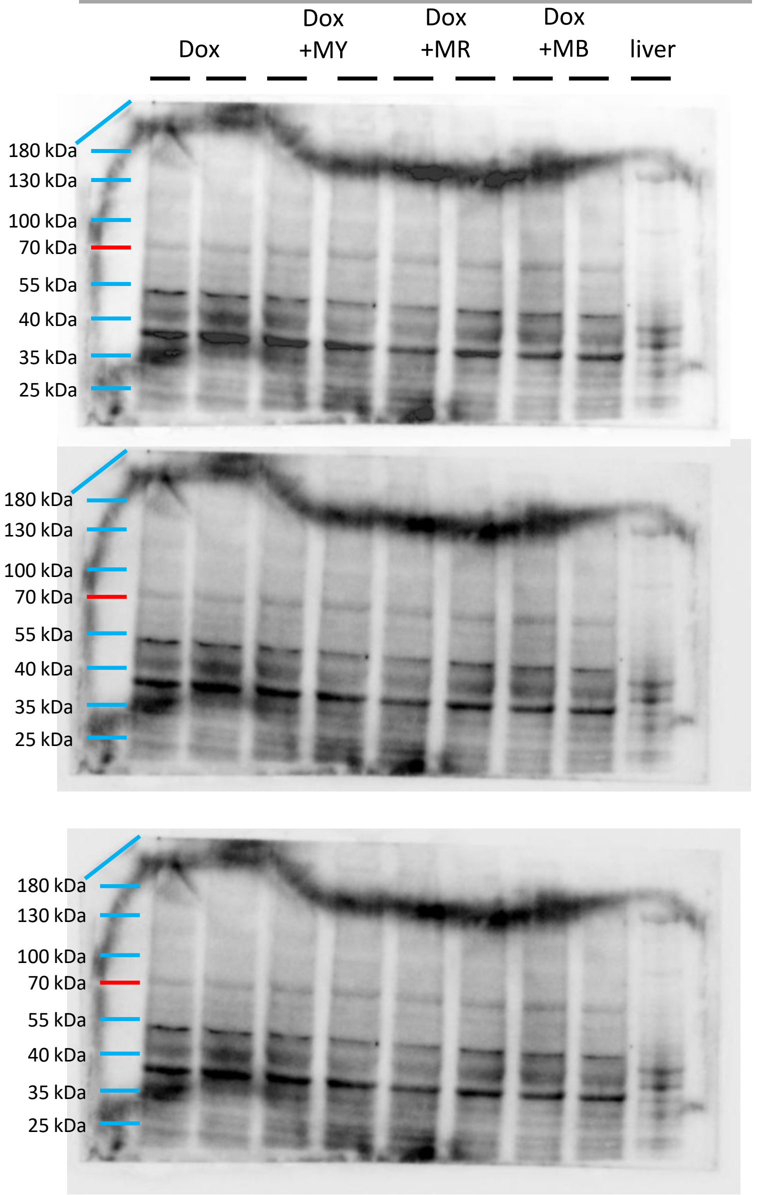

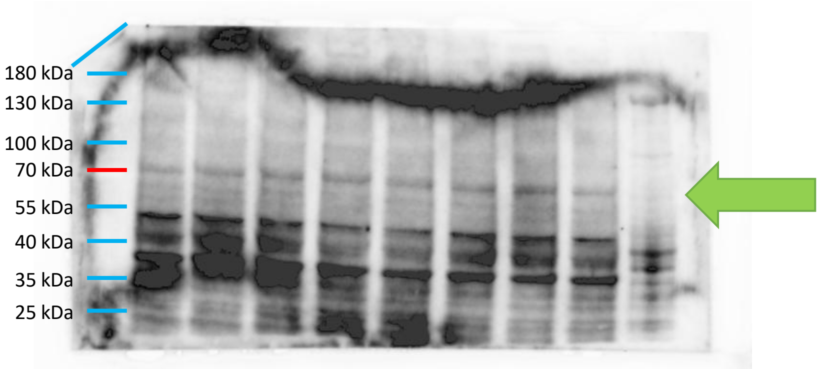

Used for Figure 3C

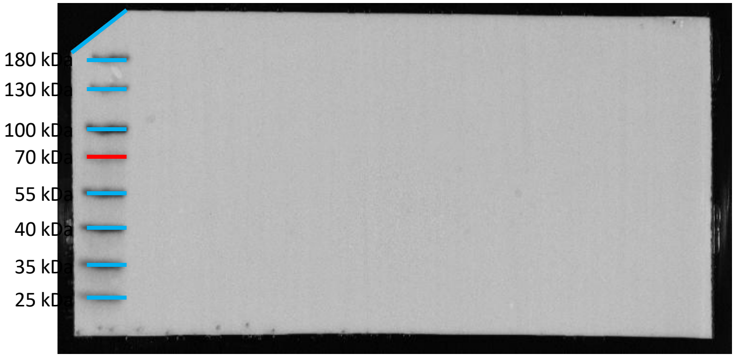

Raw WB 22Rv1

gel electrophoresis

Gel 6

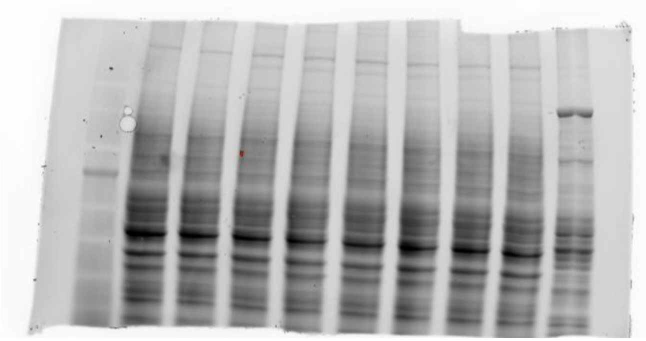

post-transfer gel

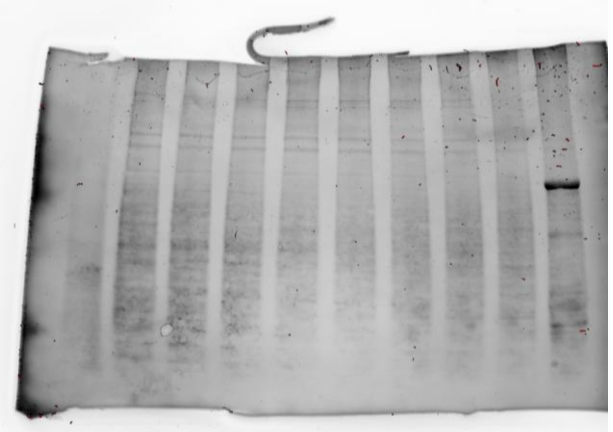

post-transfer membrane

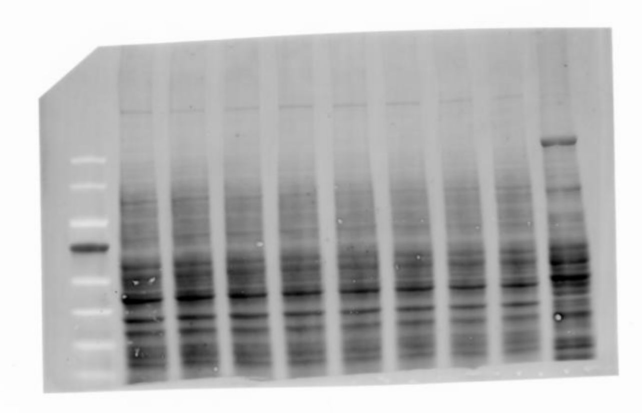

post-transfer membrane

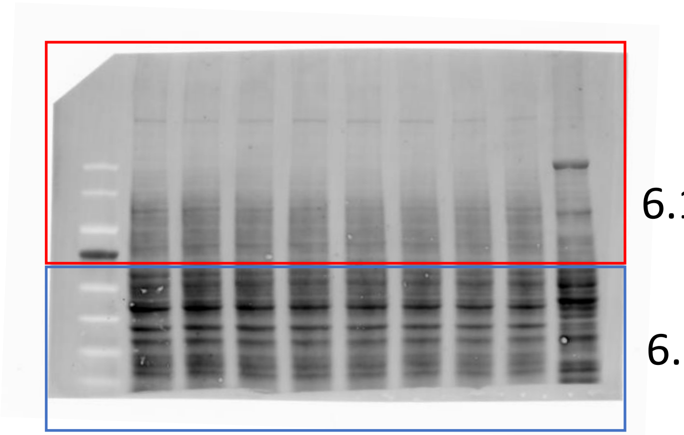

6.1

6.2

ChemiDoc image of the post-transfer membrane

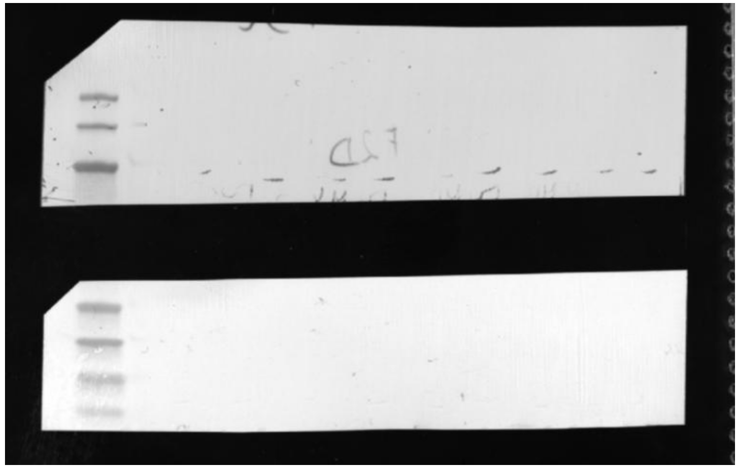

6.1

6.2

Raw WB 22Rv1

6.1 FAP anti-Rabbit

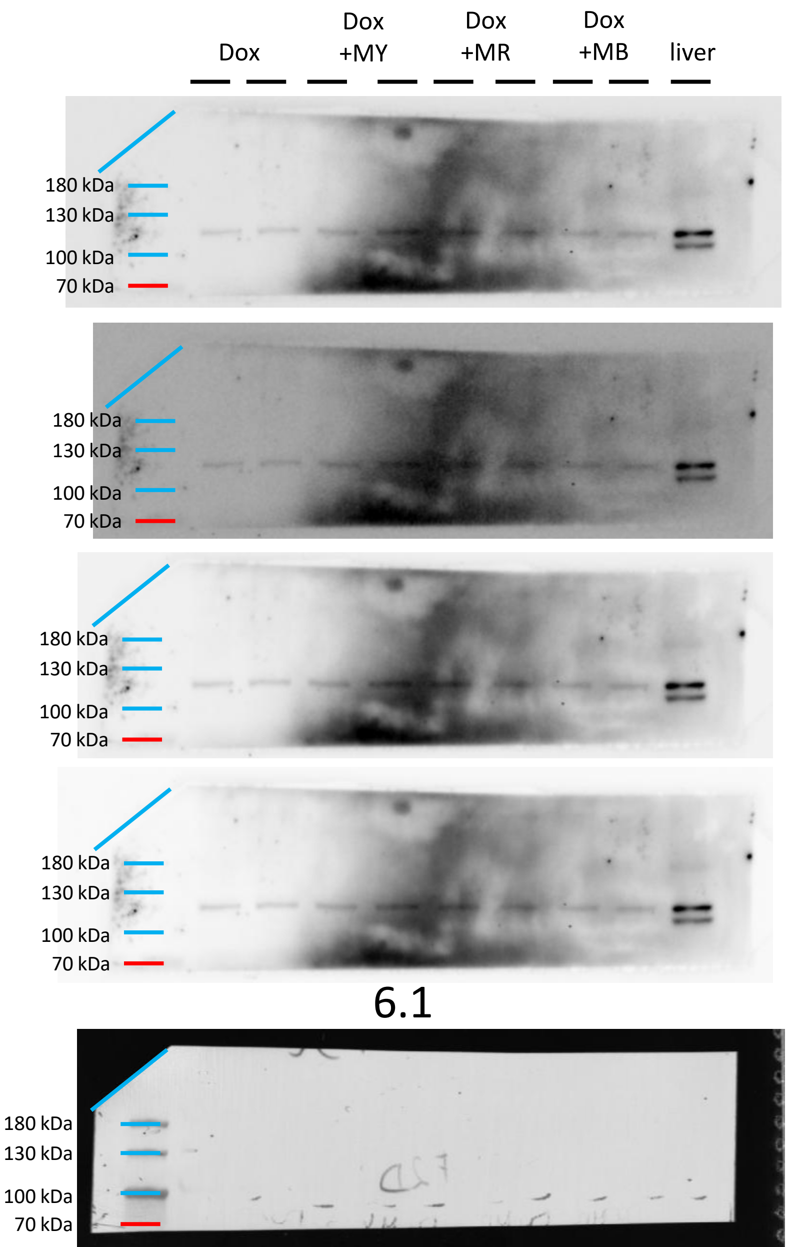

Used for Figure 3C

6.2 GAPDH anti-Mouse

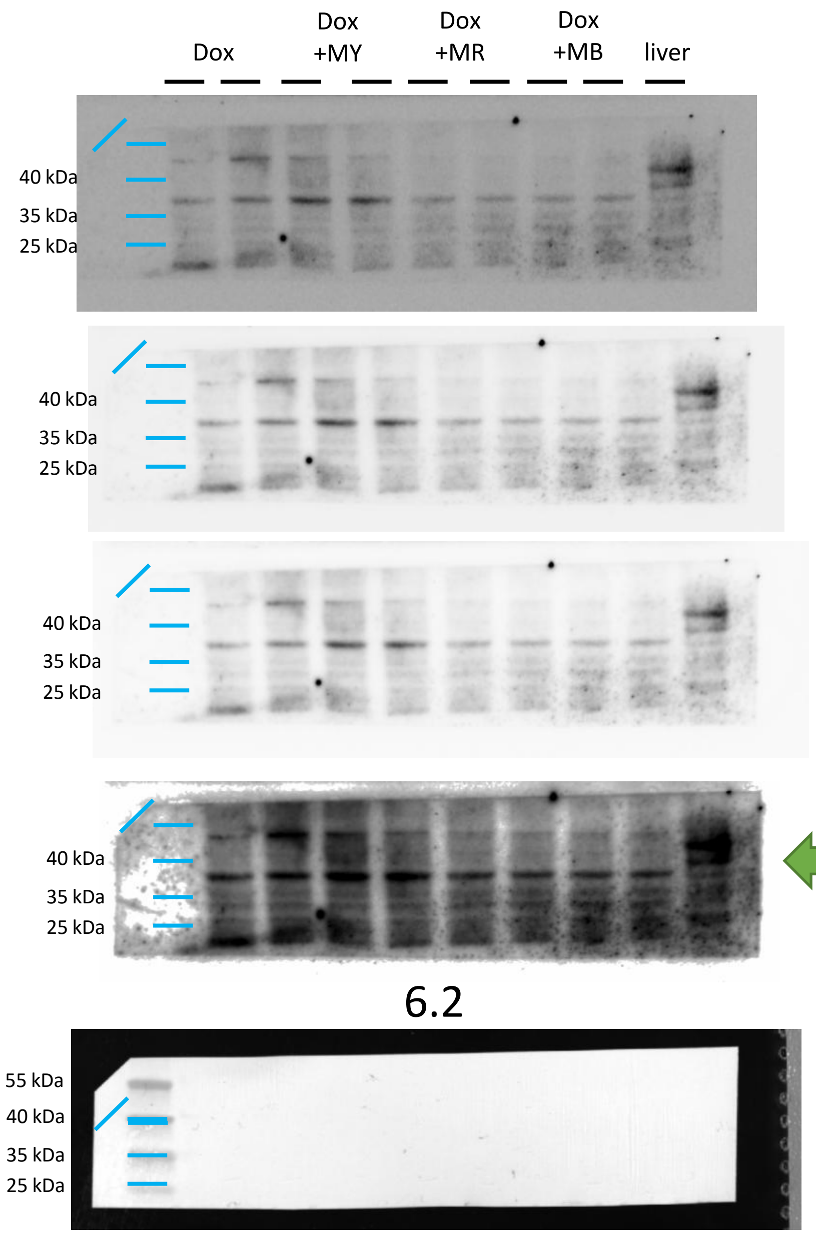

Used for Figure 2D

RAW WB co-culture 22Rv1+HDFa

Abbreviations: Liver, mouse liver tissue (positive control), DOX, doxorubicin, MY, yellow maca, MR, red maca, MB, black maca, 22Rv1, human prostate cancer cell line, HDFa, adult human dermal fibroblasts; c-cul, Co-Culture.

RAW WB co-culture 22Rv1+HDFa

Gel 1

gel electrophoresis

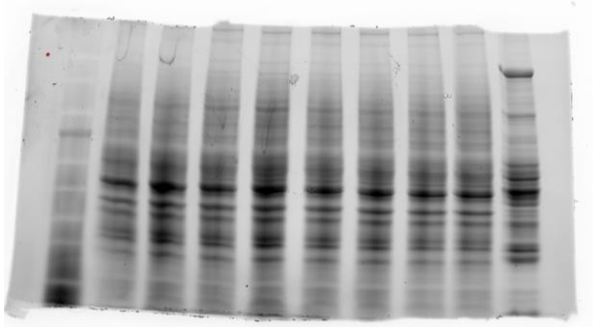

post-transfer gel

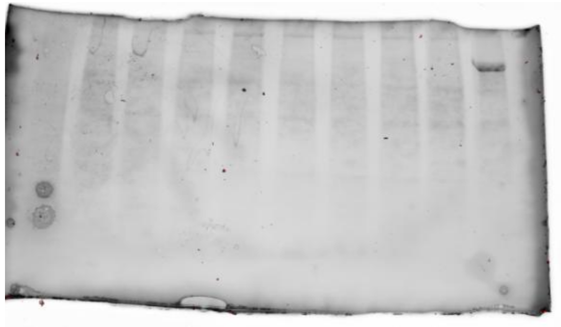

post-transfer membrane

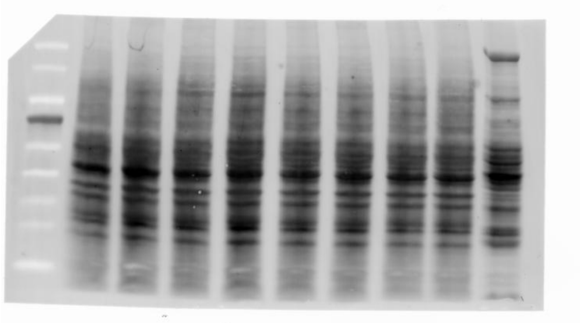

post-transfer membrane

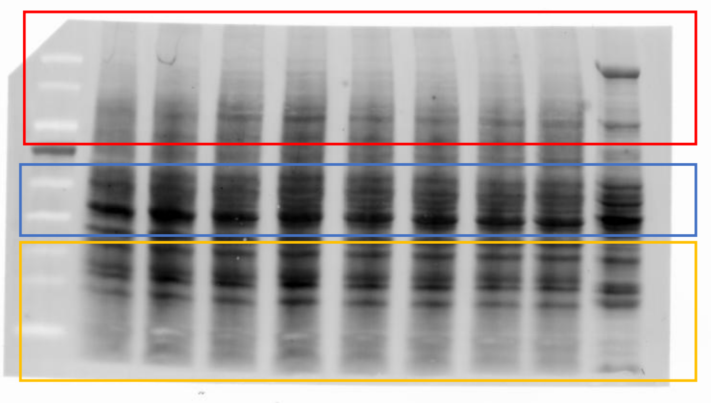

1.1 c-cul

1.2 c-cul

1.3 c-cul

ChemiDoc image of the post-transfer membrane

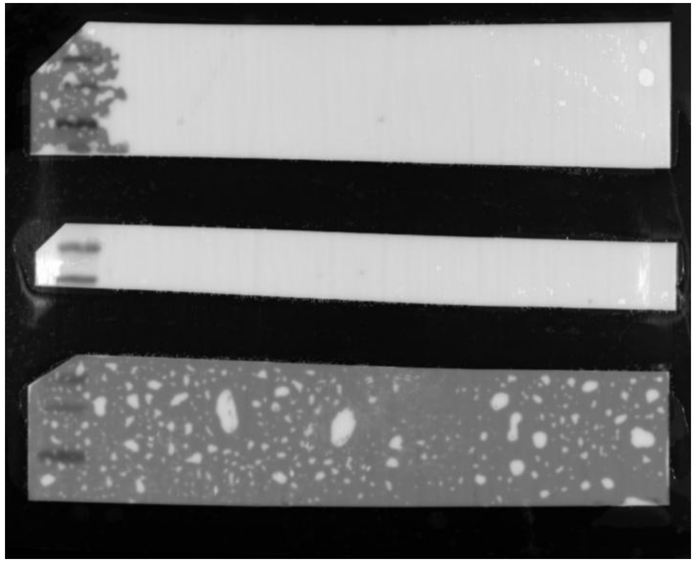

1.1 c-cul

1.2 c-cul

1.3 c-cul

RV22+HDFa 48h co-cultures; 24h 2,5 uM Dox/100 ug/ml MACA

1.1 c-cul FAP
anti-Rabbit

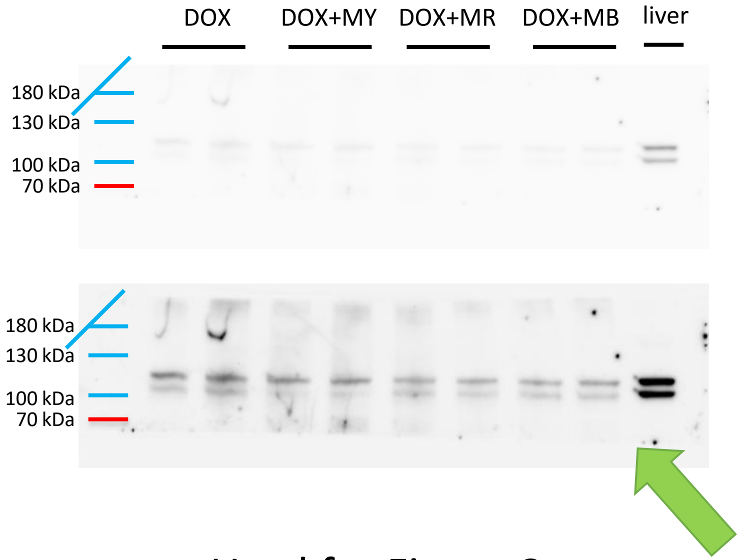

Used for Figure 8

1.2 c-cul ATGL
anti-Rabbit

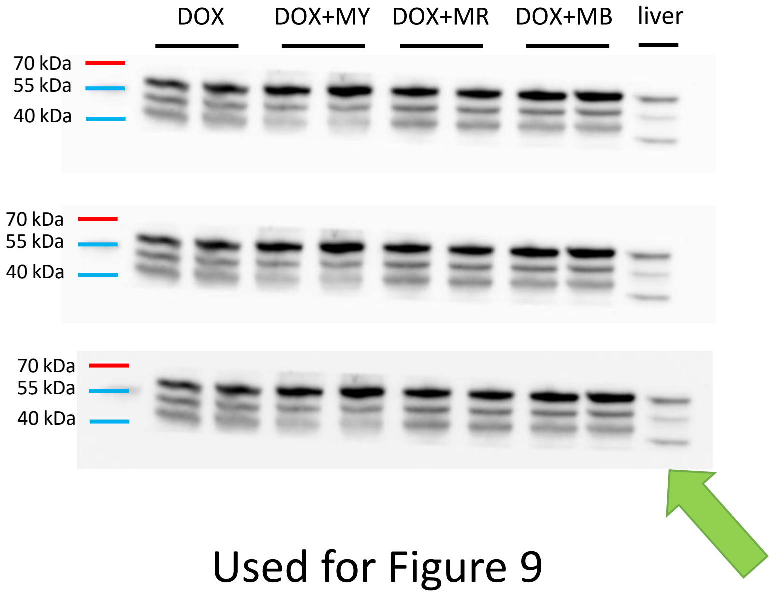

Used for Figure 9

1.3 c-cul GAPDH
anti-Mouse

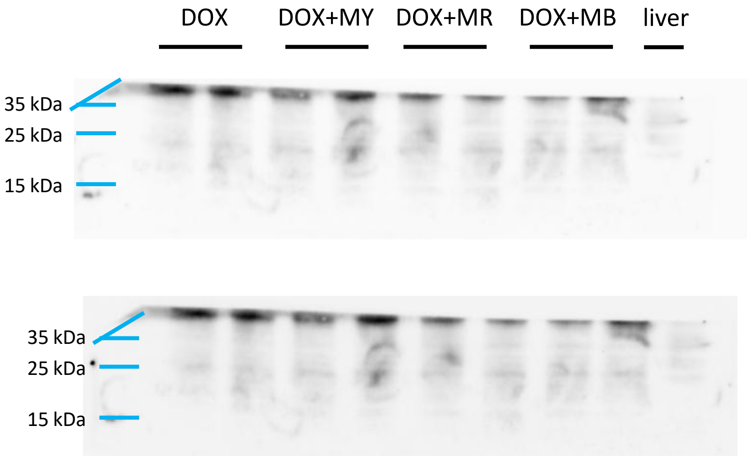

Not used

1.1 c-cul

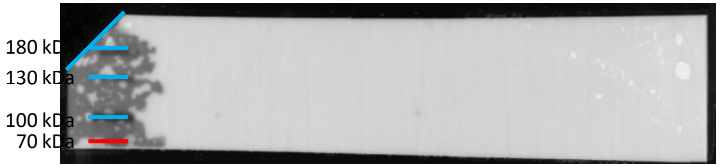

1.2 c-cul

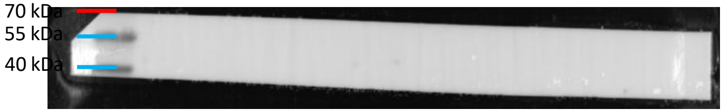

1.3 c-cul

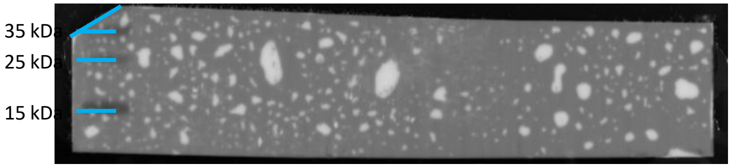

RAW WB co-culture 22Rv1+HDFa

Gel 2

gel electrophoresis

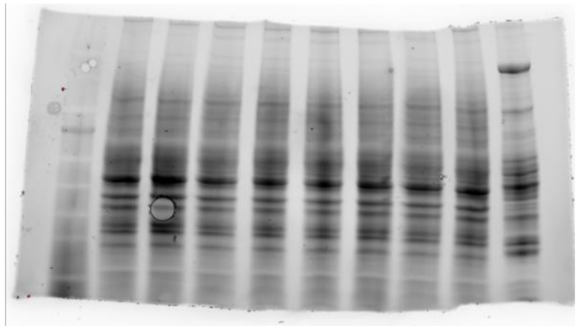

post-transfer gel

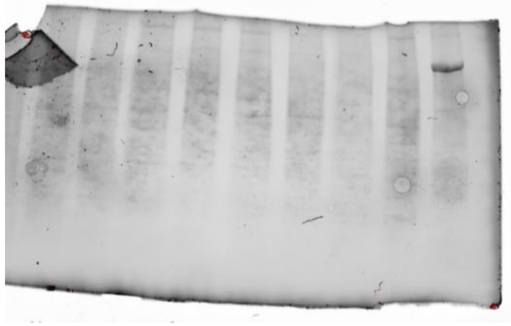

post-transfer membrane

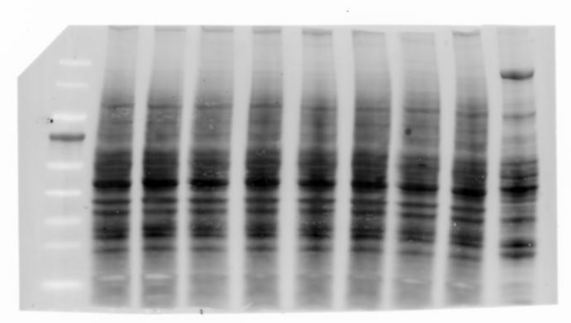

post-transfer membrane

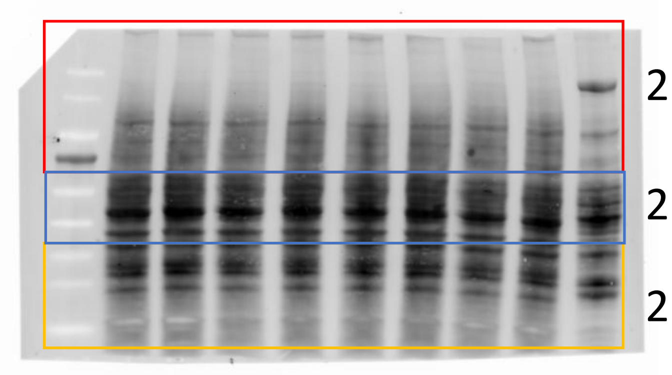

2.1 c-cul

2.2 c-cul

2.3 c-cul

ChemiDoc image of the post-transfer membrane

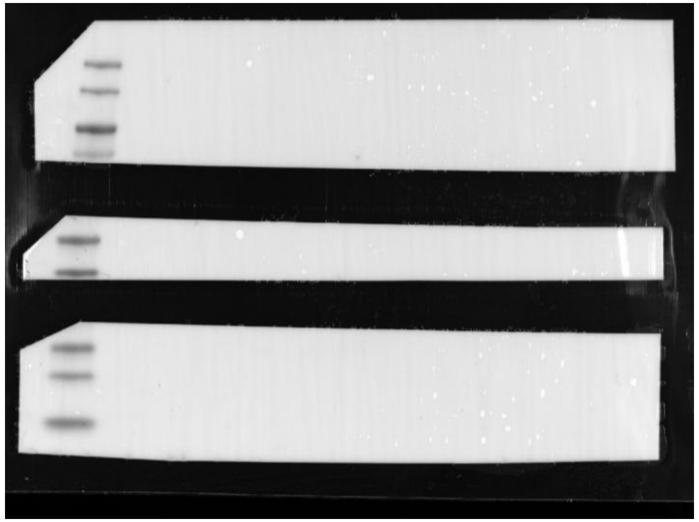

2.1 c-cul

2.2 c-cul

2.3 c-cul

RV22+HDFa 48h co-cultures; 24h 2,5 uM Dox/100 ug/ml MACA

2.1 c-cul FOXO1 anti-Rabbit

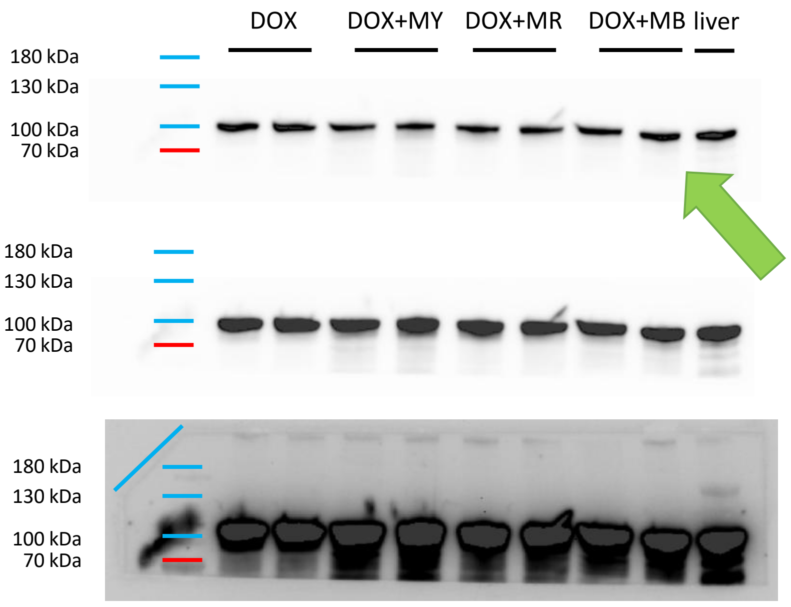

Used for Figure 8

2.1 c-cul PLIN3 anti-Mouse

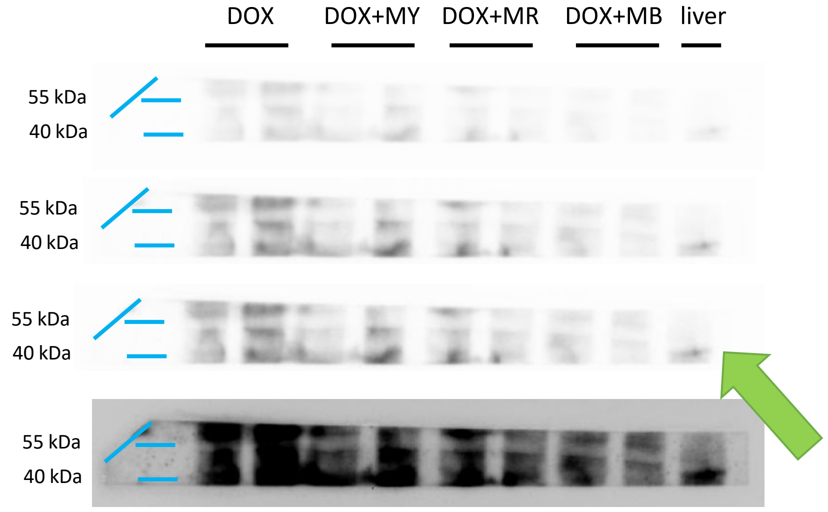

Used for Figure 9

2.3 c-cul 2 CASP3 anti-Rabbit

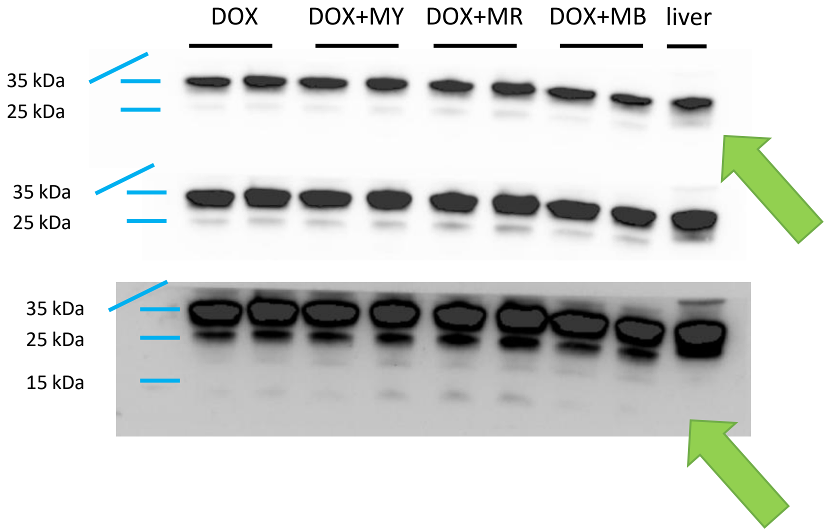

Used for figure 7F

2.1 c-cul

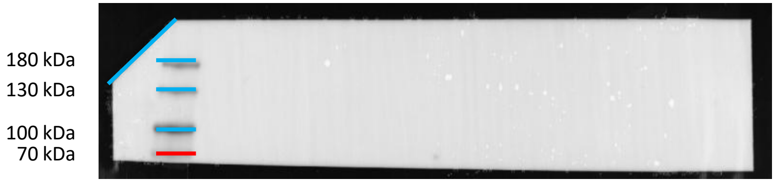

2.2 c-cul

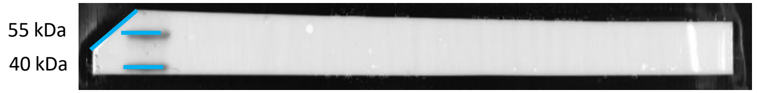

2.3 c-cul

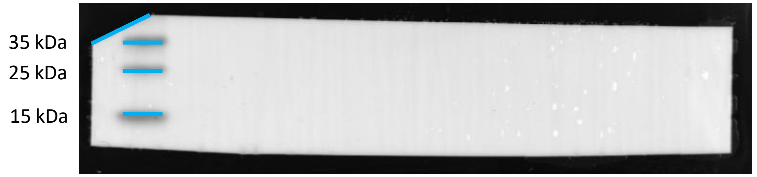

RAW WB co-culture 22Rv1+HDFa

Gel 3

gel electrophoresis

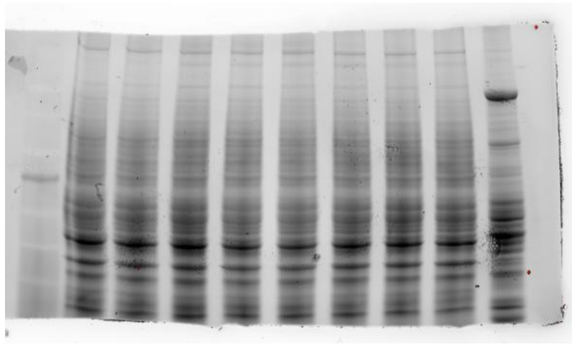

post-transfer gel

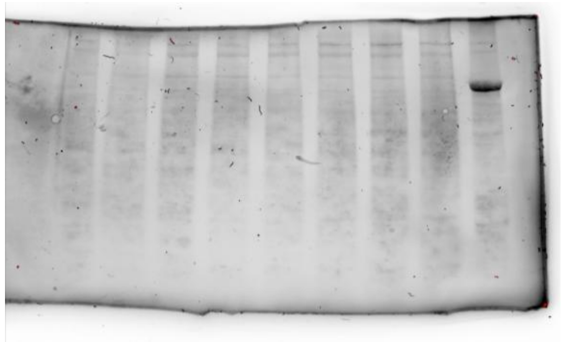

post-transfer membrane

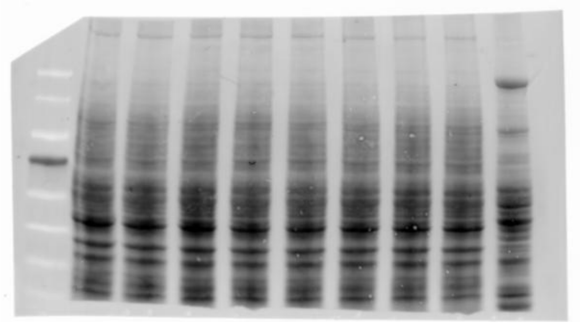

post-transfer membrane

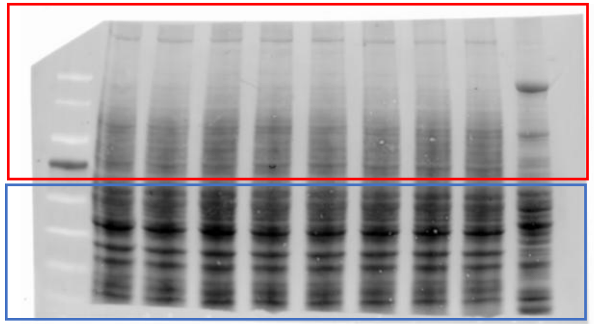

ChemiDoc image of the post-transfer membrane

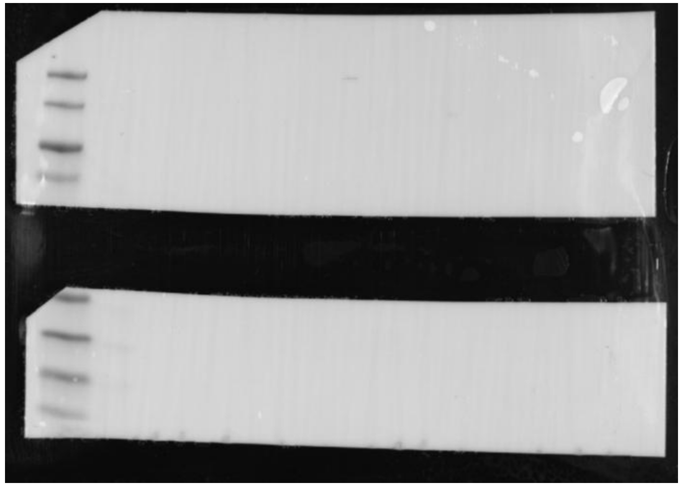

RV22+HDFa 48h co-cultures; 24h 2,5 uM Dox/100 ug/ml MACA

3.1 c-cul PSMA anti-Mouse

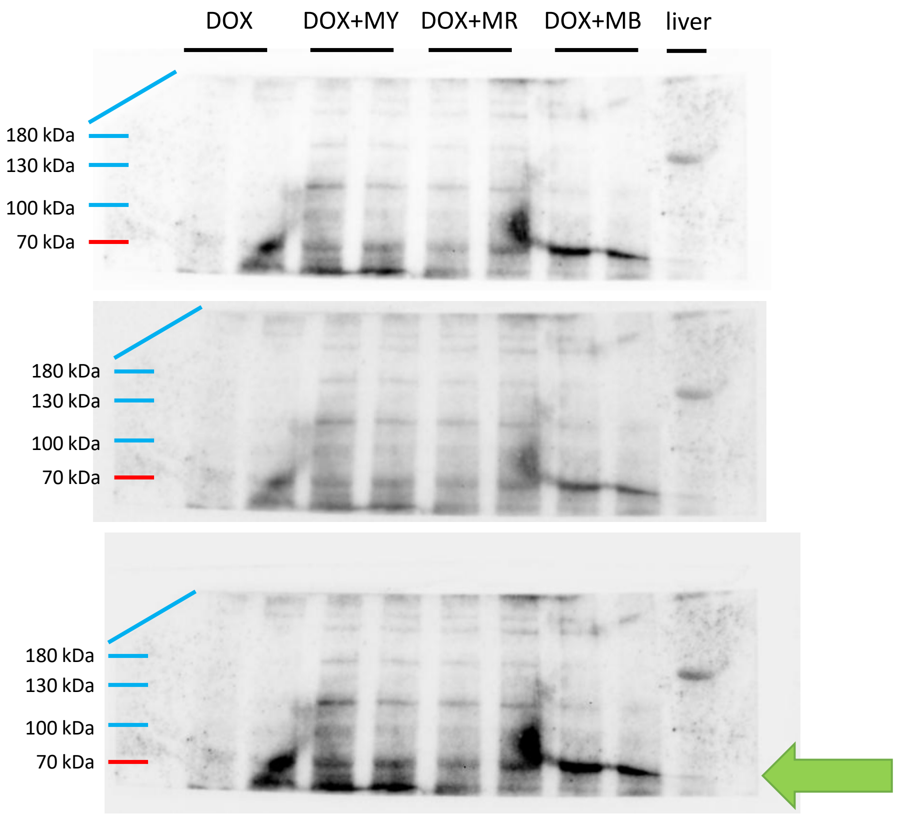

Used for Figure 8

3.1 c-cul

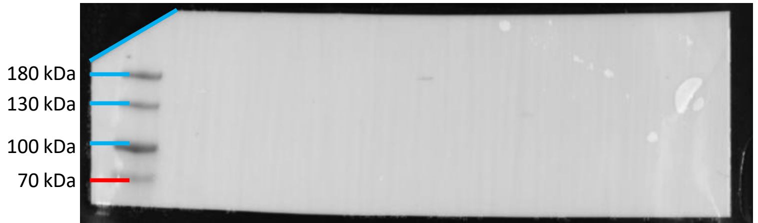

3.2 c-cul GAPDH anti-Mouse

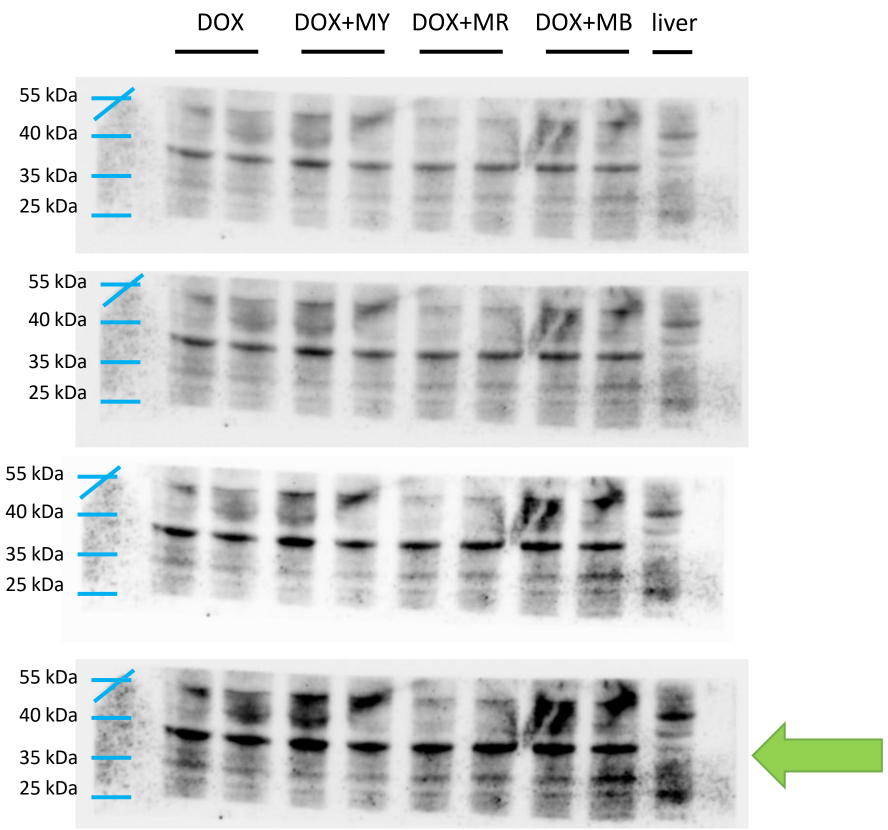

Used for Figure 7F/9

3.2 c-cul

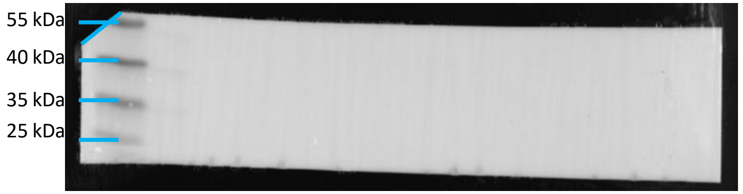

RAW WB co-culture 22Rv1+HDFa

Gel 4

gel electrophoresis

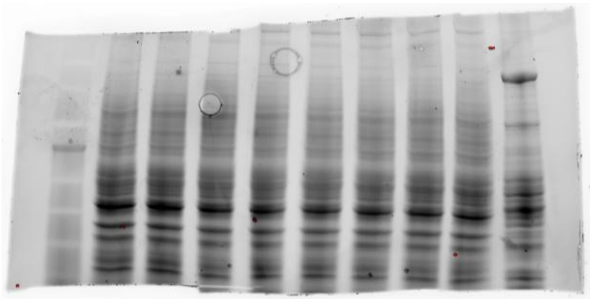

post-transfer gel

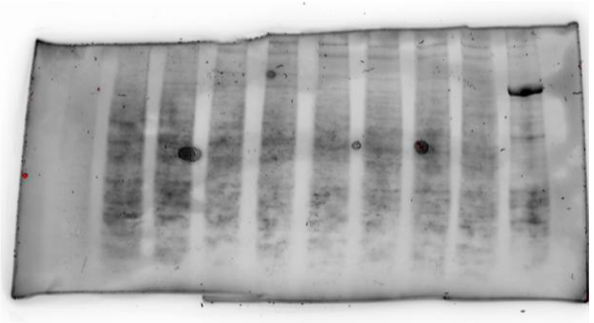

post-transfer membrane

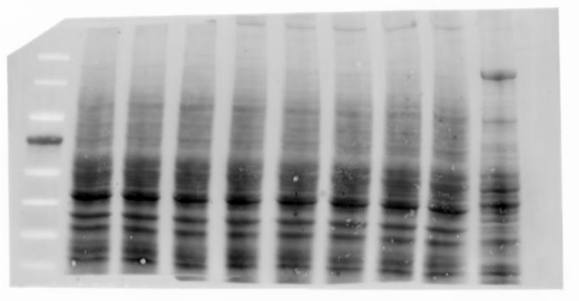

post-transfer membrane

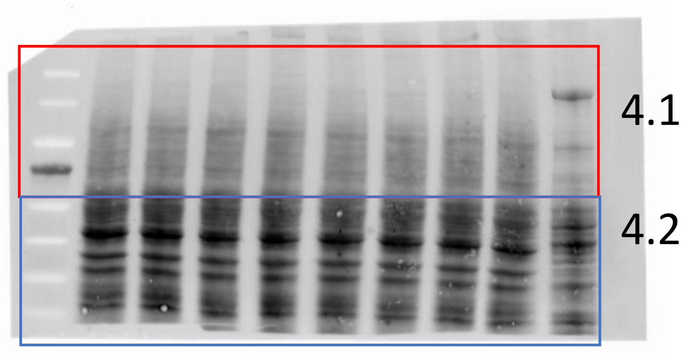

4.1 c-cul

4.2 c-cul

ChemiDoc image of the post-transfer membrane

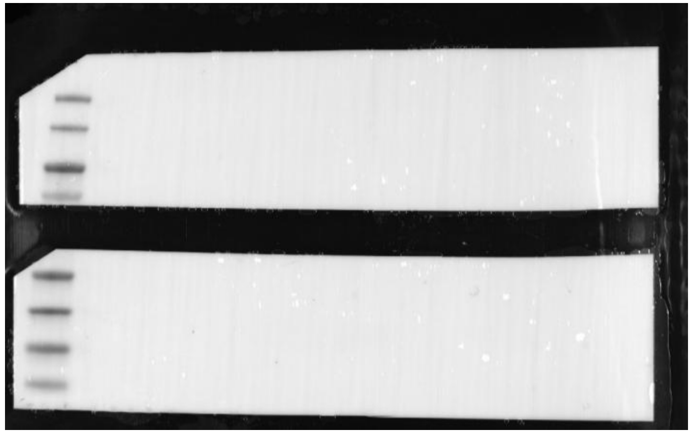

4.1 c-cul

4.2 c-cul

RV22+HDFa 48h co-cultures; 24h 2,5 uM Dox/100 ug/ml MACA

4.1 c-cul PI3K
anti-Rabbit

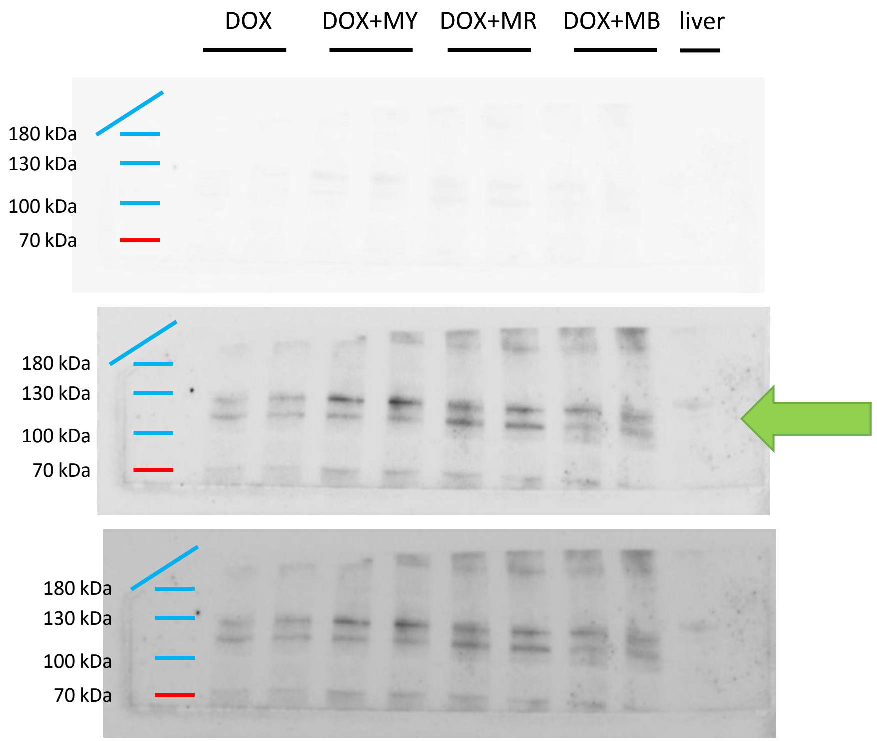

Used for Figure 8

4.2 c-cul HAT1
anti-Mouse

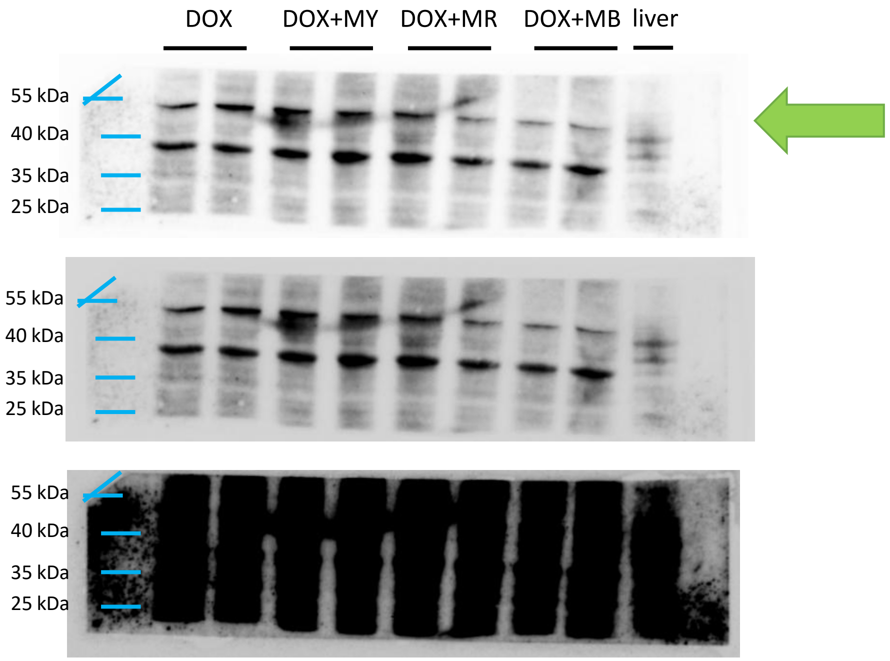

Used for Figure 8

4.1 c-cul

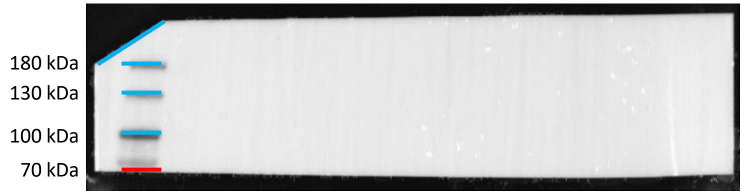

4.2 c-cul

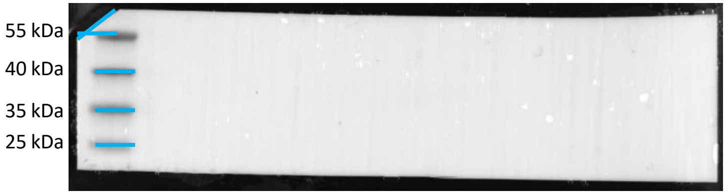

RAW WB co-culture 22Rv1+HDFa

Gel 5

gel electrophoresis

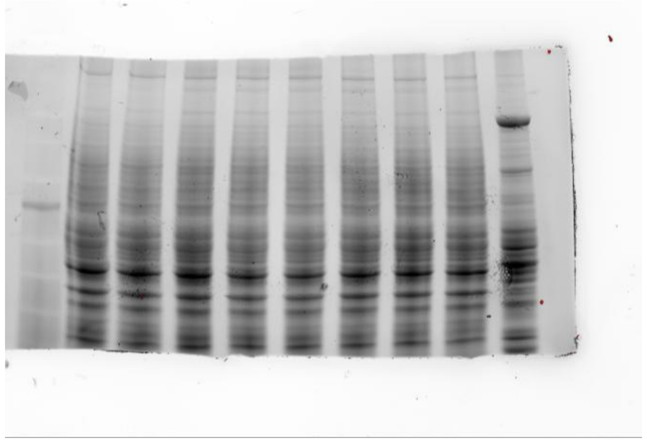

post-transfer gel

post-transfer membrane

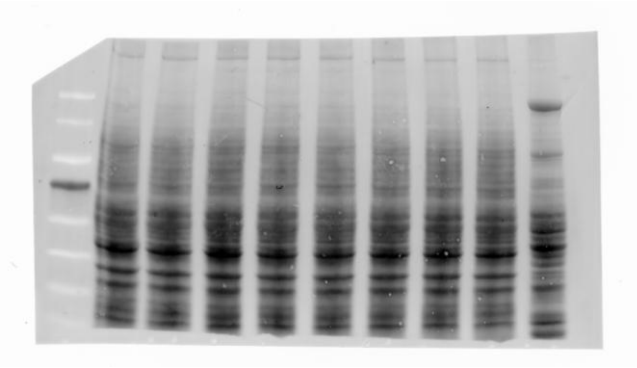

post-transfer membrane

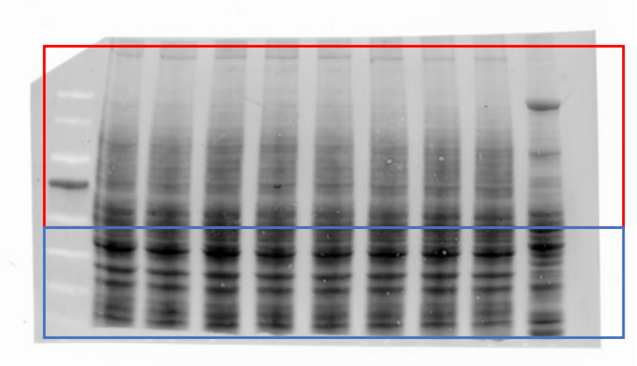

5.1 c-cul

5.2 c-cul

ChemiDoc image of the post-transfer membrane

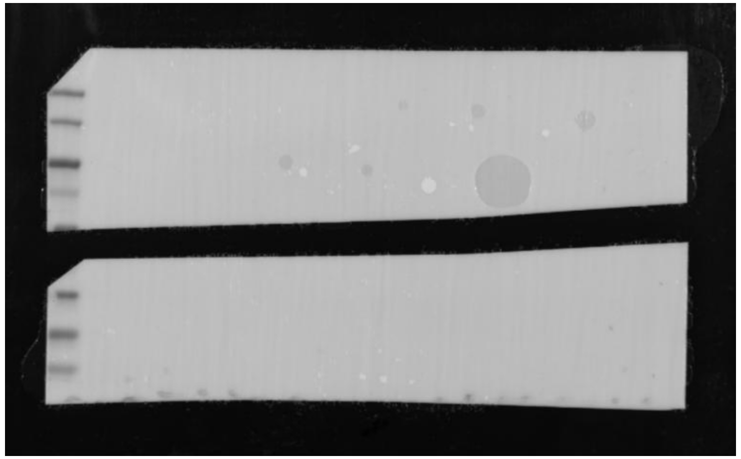

5.1 c-cul

5.2 c-cul

5.1

Not used

5.2 c-cul GAPDH anti-Mouse

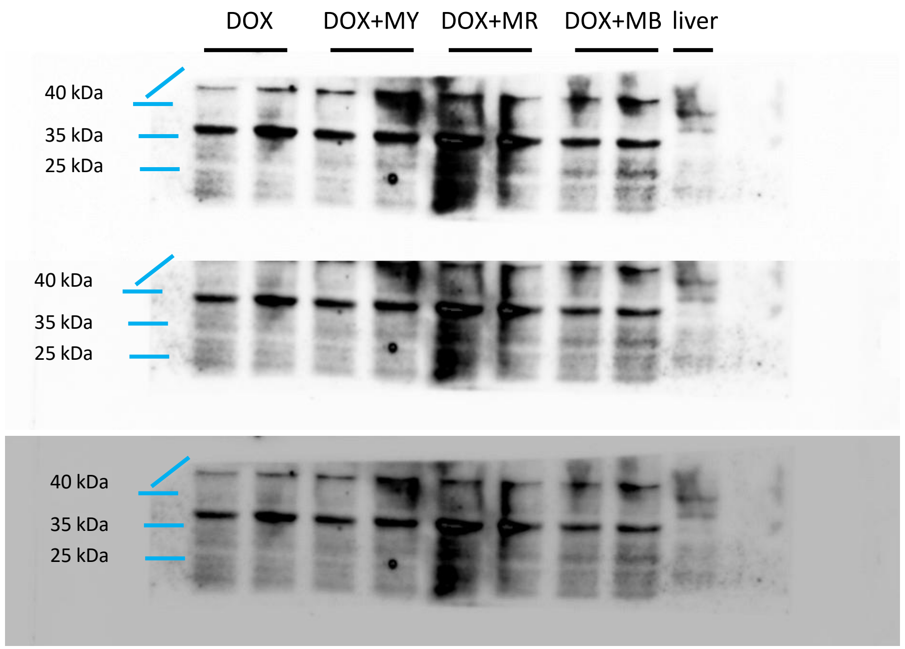

Not used

5.1 c-cul

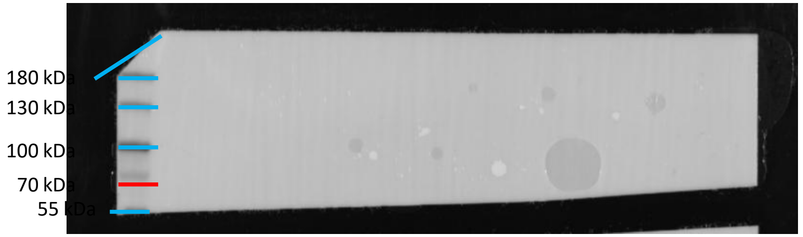

5.2 c-cul

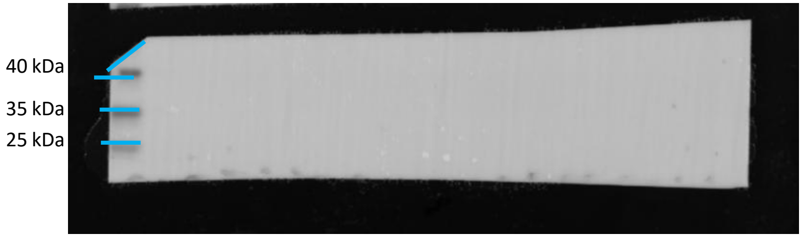

RAW WB co-culture 22Rv1+HDFa

Gel 6

gel electrophoresis

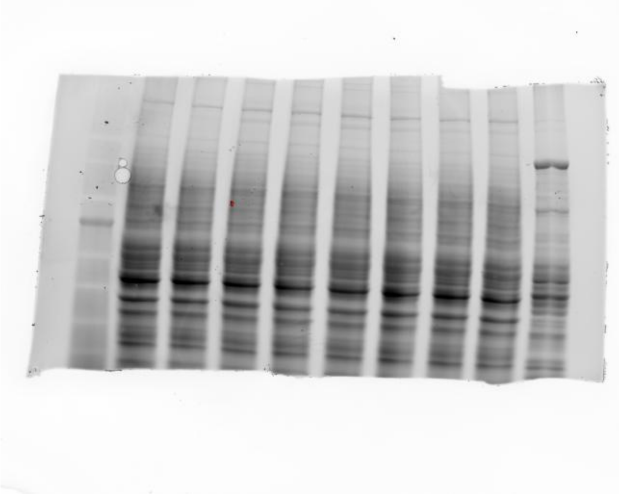

post-transfer gel

post-transfer membrane

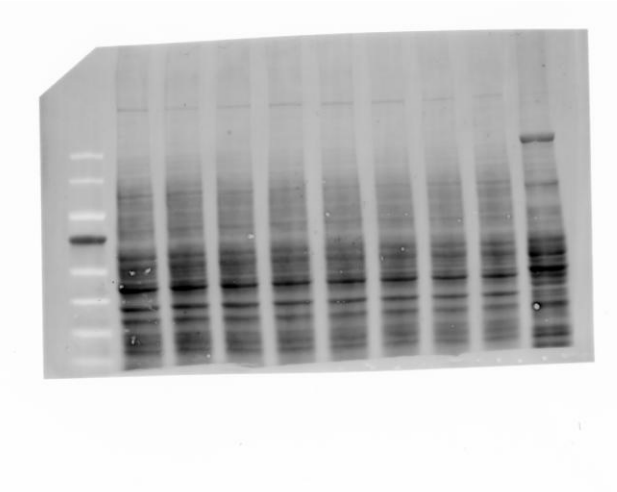

post-transfer membrane

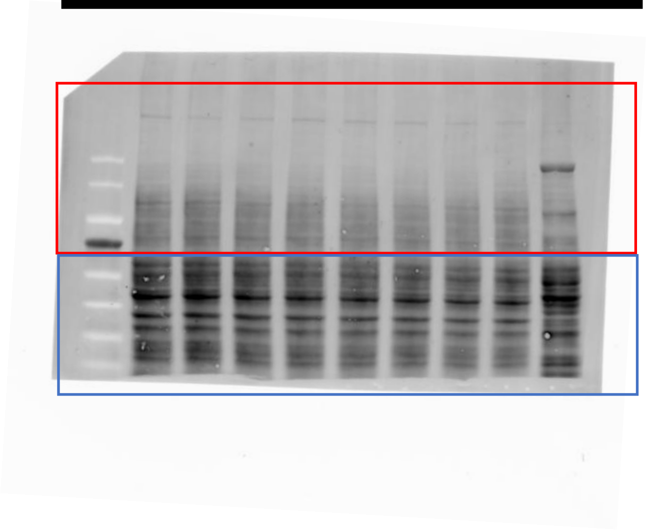

6.1 c-cul

6.2 c-cul

ChemiDoc image of the post-transfer membrane

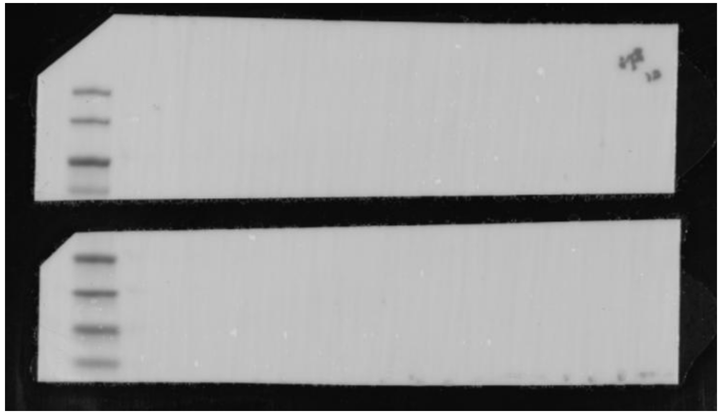

6.1 c-cul

6.2 c-cul

RV22+HDFa 48h co-cultures; 24h 2,5 uM Dox/100 ug/ml MACA

6.1

Not used

6.2 c-cul ABHD5 anti-Mouse

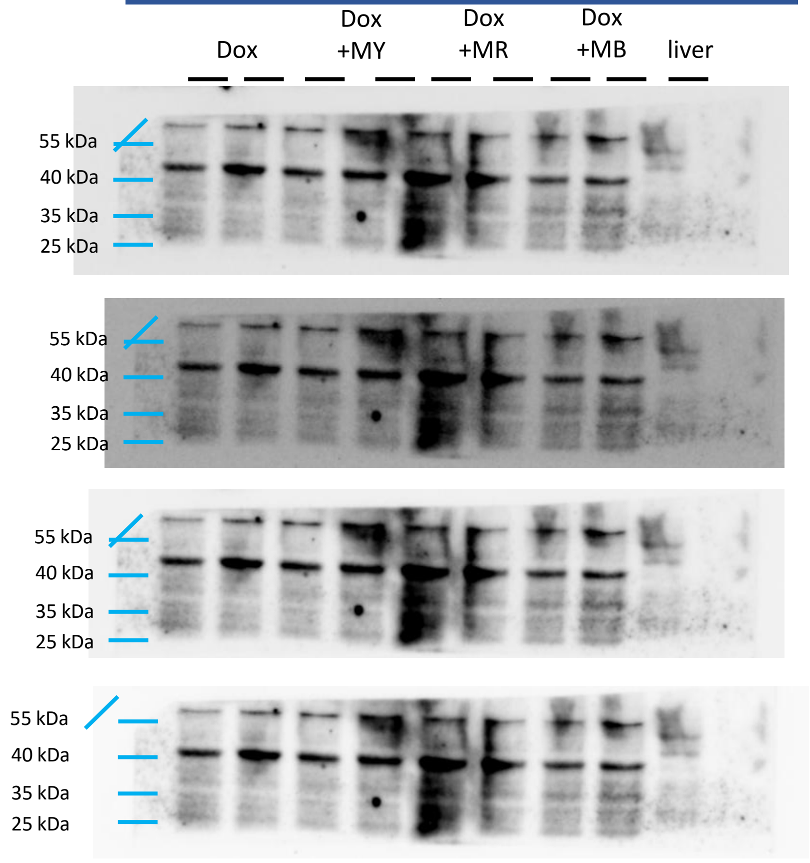

Used for Figure 9

6.1 c-cul

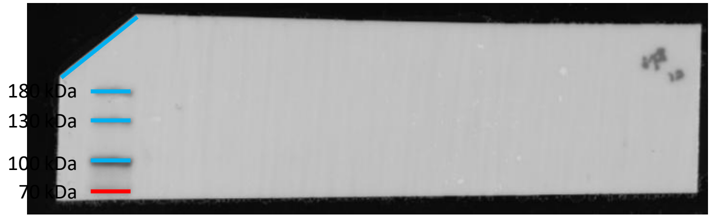

6.2 c-cul

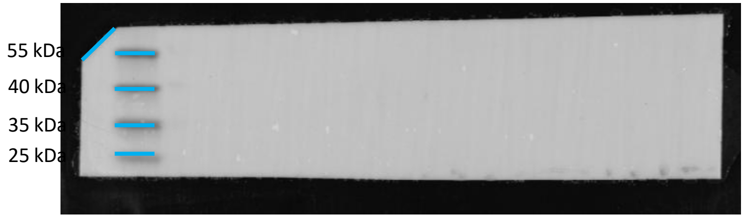

WB used for figures

Abbreviations: Liver, mouse liver tissue (positive control), DOX, doxorubicin, MY, yellow maca, MR, red maca, MB, black maca, 22Rv1, human prostate cancer cell line, HDFa, adult human dermal fibroblasts; c-cul, Co-Culture.

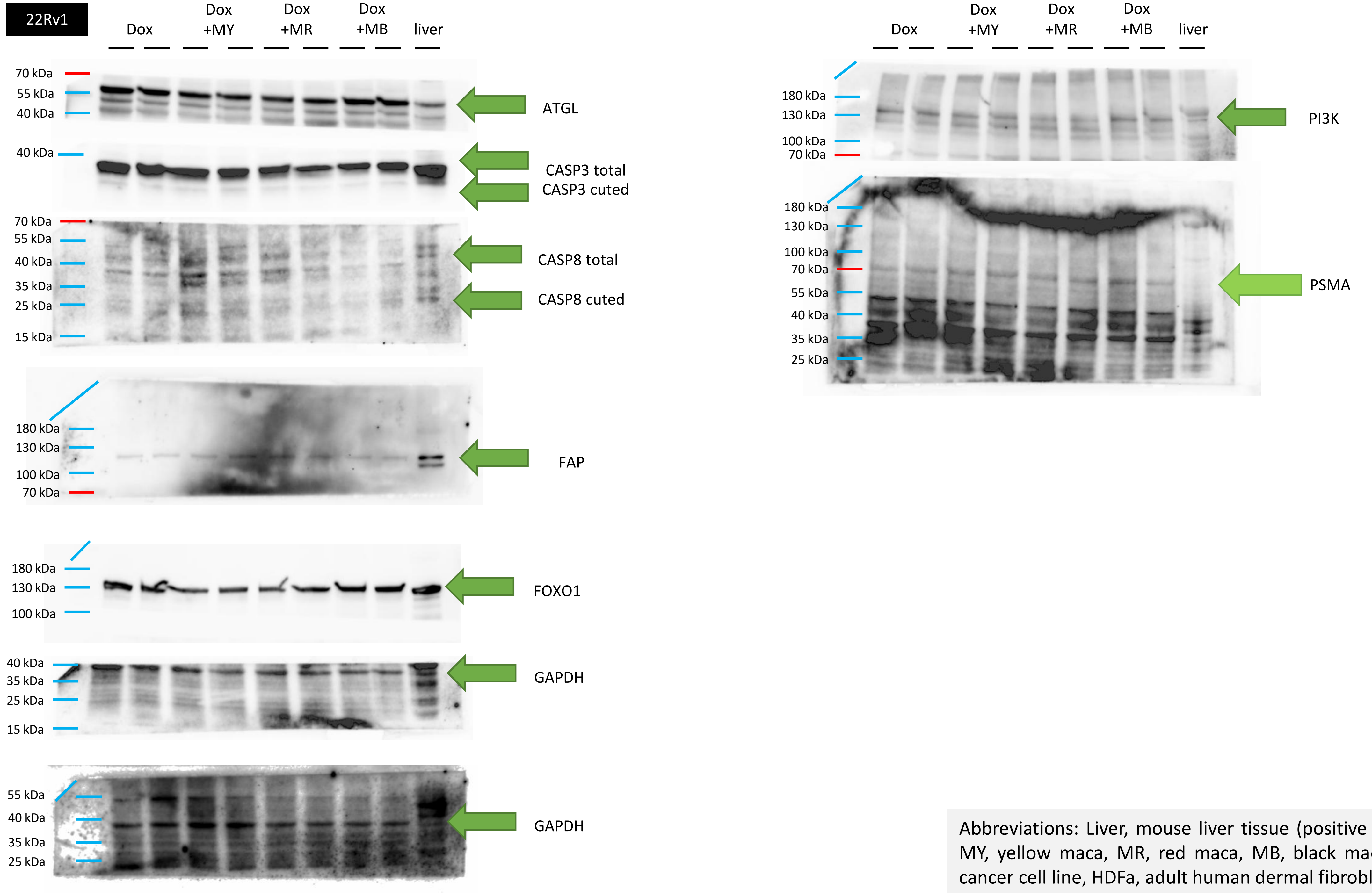

Abbreviations: Liver, mouse liver tissue (positive control), DOX, doxorubicin, MY, yellow maca, MR, red maca, MB, black maca, 22Rv1, human prostate cancer cell line, HDFa, adult human dermal fibroblasts; c-cul, Co-Culture.

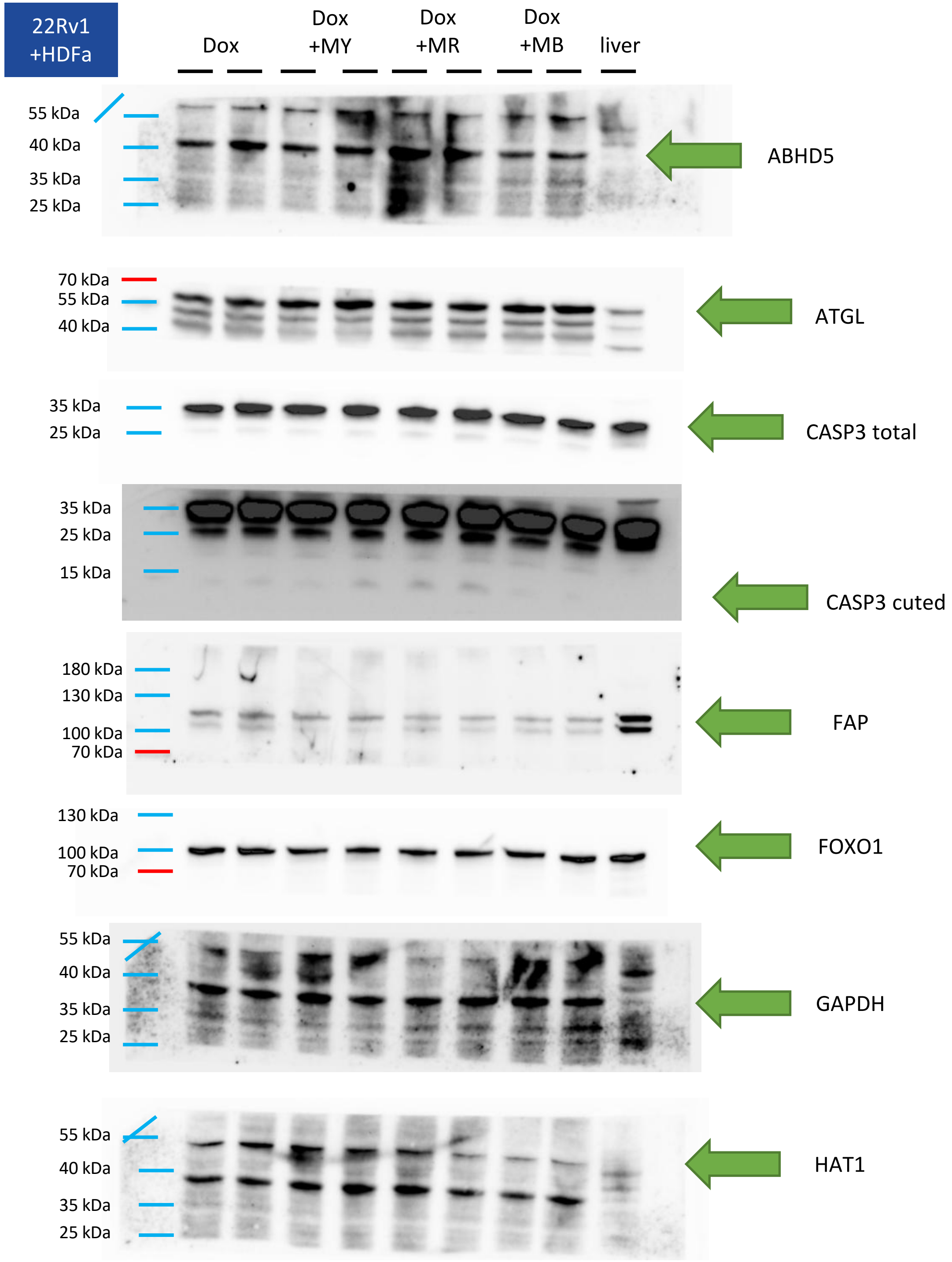

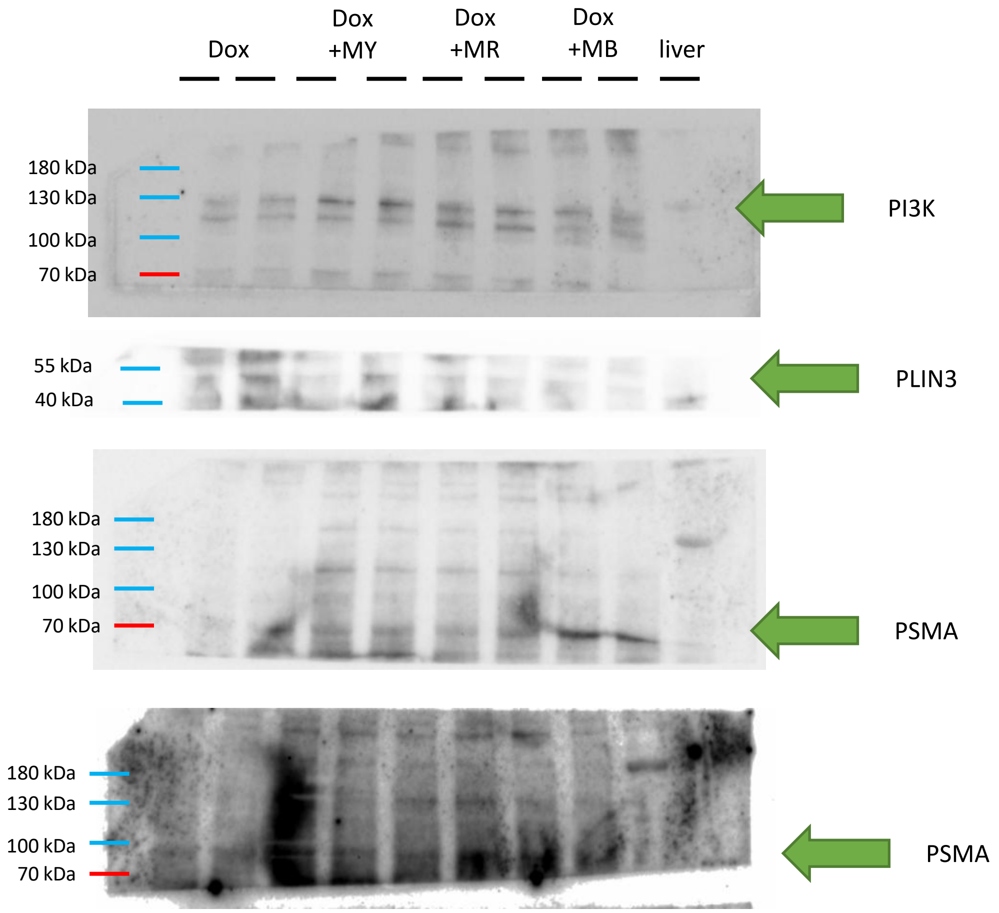

Abbreviations: Liver, mouse liver tissue (positive control), DOX, doxorubicin, MY, yellow maca, MR, red maca, MB, black maca, 22Rv1, human prostate cancer cell line, HDFa, adult human dermal fibroblasts; c-cul, Co-Culture.
